# Health quality management practises for cardiovascular diseases, diabetes, and obesity care in the UAE: a scoping review

**DOI:** 10.3389/fpubh.2025.1703376

**Published:** 2026-01-12

**Authors:** Zufishan Alam, Kanika Sinha, Fatima AlBedwawi, Hanin Osman, Md. Hafizur Rahman, Fadumo Noor, Nazik Nurelhuda

**Affiliations:** School of Health Sciences, Hamdan Bin Mohammed Smart University, Dubai, United Arab Emirates

**Keywords:** cardiovascular disease, diabetes, Donabedian model, effectiveness, healthcare, Institute of Medicine quality framework, non communicable chronic diseases, obesity

## Abstract

**Background:**

Non-communicable diseases (NCDs), such as cardiovascular diseases (CVD), diabetes, and obesity, are increasingly prevalent in the United Arab Emirates (UAE), driven by lifestyle changes and demographic shifts. This review maps the current research landscape on health quality management (HQM) practises for the three NCDs in the UAE, uniquely analysed through health quality frameworks.

**Methods:**

A scoping review of UAE-based studies and grey literature on HQM for the three NCDs (2015–2025), following the Arksey and O’Malley framework and in alignment with the Preferred Reporting Items for Systematic Reviews and Meta-Analyses extension for Scoping Reviews (PRISMA-ScR) guidelines, was conducted. A subsequent focused two-dimensional analysis of interventional studies assessed health quality improvement efforts, incorporating guideline documents and reports for policy context. Findings were analysed using the Institute of Medicine’s (IOM) six quality domains and the Donabedian model (structure, process, outcomes).

**Results:**

Of the identified 549 relevant records, observational studies were predominant, with the majority conducted in larger urban Emirates and focusing on CVD and diabetes. The interventional studies and grey literature documents in the subset analysis largely addressed the effectiveness domain of HQM. Most interventions focused on lifestyle modification through education, digital tools, and multidisciplinary support, with modest clinical improvements observed in most studies. However, study designs were often limited by small sample sizes, the lack of control groups, short durations, and methodological variability. Policy documents emphasised structure and process with limited focus on outcome monitoring.

**Conclusion:**

Although intervention research addressing NCDs in the UAE shows promise, it remains methodologically limited and inconsistently distributed across the six HQM domains, with a heavy emphasis on effectiveness and a relative underrepresentation of equity, efficiency, and timeliness, as noted in the reviewed literature. There is a pressing need to implement standardised quality indicators addressing all HQM domains, strengthen cross-sector collaboration, and integrate patient perspectives to translate research progress into equitable, sustainable improvements in NCD care. By integrating health quality frameworks, this review provides a foundation for strengthening NCD care standards in the UAE.

## 1 Introduction

Non-communicable diseases (NCDs), such as cardiovascular diseases (CVDs), diabetes, and obesity, pose a significant public health challenge worldwide, causing significant mortality and morbidity (1). However, their impact is particularly severe in the United Arab Emirates (UAE), where the prevalence of these chronic conditions has reached alarming levels in recent years (2).

CVDs are the leading cause of death in the UAE, accounting for approximately 40% of all deaths nationally (2). As of 2024, the age-adjusted prevalence of people living with diabetes in the UAE, as per the International Diabetes Federation, is 20.7% (3). In addition, 27.8% are classified as obese, with 67.9% of adults being overweight (4). These conditions impose a substantial burden not only on the health of the population but also on the national economy. In 2019, the total economic burden of NCDs in the UAE was estimated at AED 39.9 billion, of which two-thirds (AED 26 billion) was attributed to CVD alone (5). Accordingly, this study focuses on these three conditions.

The interconnection between CVD, diabetes, and obesity is well established, with obesity being a major risk factor for both CVD and type 2 diabetes (6). A large meta-analysis as part of the “Emerging Risk Factors Collaboration” studies found that diabetes accounts for 11% (10–12%) of cardiovascular deaths (7). In addition to biological factors, certain modifiable behavioural risk factors are common amongst these NCDs, such as unhealthy dietary patterns, physical inactivity, and tobacco use (8). These, in turn, are influenced by wider determinants of health such as socioeconomic status, rapid urbanisation, cultural beliefs, sedentary lifestyles, and dietary transitions, which have contributed to the NCD burden across the region’s diverse population (9). In this context, prevention and early management approaches often converge across the three conditions, given their overlapping determinants and risk profiles.

The UAE’s healthcare system follows a mixed governance model in which the federal Ministry of Health and Prevention (MOHAP) sets national policies and standards, whilst Emirate-specific authorities such as the Department of Health—Abu Dhabi (DOH) and the Dubai Health Authority (DHA) oversee services within their Emirates. It is a mixed public–private system with the private sector playing a leading role in service delivery. It has near-universal population coverage, with government-funded programmes such as Thiqa in Abu Dhabi (10) and Saada in Dubai (11) providing comprehensive coverage for citizens, whilst employers are mandated to provide health insurance for expatriate residents and their dependents (12, 13). In this scoping review, the term expatriate refers to non-UAE citizens residing in the country on employment- or family-based residence visas.

Health management, as defined by the European Health Management Association, is “the practice of providing guidance and leadership to promote and support health at the individual, organisational and systemic levels” (14). This concept embraces a holistic vision of health that recognises the influence of behavioural, social, and environmental determinants. Health quality management (HQM), as defined by the Institute of Medicine (IOM), encompasses six domains: safety, effectiveness, patient-centeredness, timeliness, efficiency, and equity (15). Together, these domains offer a comprehensive framework for evaluating NCD care. The framework is further strengthened by the Donabedian model, which assesses healthcare quality through three foundational components: structure (healthcare infrastructure and organisational characteristics), process (care delivery activities and procedures), and outcome (effects of care on patient and population health) (16). Collectively, these perspectives offer an integrated approach to understanding and improving the quality of NCD care. Despite the UAE’s prioritisation of NCDs and notable investments in healthcare infrastructure, persistent gaps in HQM remain (17).

No review to date has focused on UAE-based interventional studies or mapped them against quality standards. By examining the extent to which the domains are addressed in CVD, diabetes, and obesity management in the UAE, the current strengths, gaps, and opportunities for advancing HQM can be identified. Therefore, this review aimed to scope the current research landscape for the health management of the three NCDs—CVD, diabetes, and obesity—in the UAE and to conduct a focused assessment of HQM practises by examining interventional studies and frameworks (95).

## 2 Methods

We conducted this scoping review following the Arksey and O’Malley framework (18) and in alignment with the Preferred Reporting Items for Systematic Reviews and Meta-Analyses extension for Scoping Reviews (PRISMA-ScR) guidelines (refer to Supplementary Table 1 for the checklist) (19). A scoping review approach was selected, given the heterogeneity of study designs, populations, and outcome measures in UAE NCD research. Unlike a systematic review, which focuses on specific interventions or outcomes, a scoping review enables mapping the breadth and characteristics of available evidence, identifying conceptual and methodological gaps, and informing priorities for future research (20).

The methodology consisted of five core stages, including identifying the research question, identifying relevant records, selecting the records, charting the data, and collating, summarising, and reporting the results (18).

### 2.1 Search strategy and data sources

A comprehensive search was undertaken across three electronic databases: PubMed, Scopus, and ProQuest, from 1 January 2015 to 1 March 2025. We focused on the last 10 years to capture recent evidence, reflecting the rapidly evolving healthcare landscape within the UAE. We used a combination of MeSH terms and free-text keywords, integrated using Boolean operators, to ensure comprehensive retrieval of relevant literature. The search string incorporated three concepts: (1) the NCDs (cardiovascular disease OR diabetes OR obesity), (2) terms related to quality of care or health management, and (3) the UAE context. The search string included all UAE Emirates (Abu Dhabi, Dubai, Sharjah, Ajman, Ras Al Khaimah, Fujairah, and Umm Al Quwain) as well as relevant risk factors for the three NCDs (detailed search strategy with specific terms and combinations used in PubMed is provided in Supplementary Table 1).

To complement the academic literature, we conducted a grey literature search from the websites of relevant multisectoral national organisations (both governmental and non-governmental), namely the Ministry of Health and Prevention (MOHAP), Dubai Health Authority (DHA), Department of Health Abu Dhabi (DOH), Emirates Health Services (EHS), Abu Dhabi Public Health Centre (ADPHC), Community Development Authority, Ministry of Community Empowerment, Mohammed Bin Rashid Al Maktoum Knowledge Foundation, Abu Dhabi Early Childhood Authority, Emergency, Crises, and Disaster Management Centre Abu Dhabi, Family Care Authority, National Health Insurance Company—UAE, Statistics Centre Abu Dhabi, Sharjah Health Authority, Abu Dhabi Health Services Company, Dubai Health, Pure Health, Emirates Drug Establishment, Emirates Cardiac Society, AlMubarakah Foundation, and the Institute for Healthy Living Abu Dhabi.

We also searched the websites of international organisations, namely World Health Organisation (WHO), United Nations International Children’s Emergency Fund (UNICEF), International Diabetes Foundation (IDF), American Heart Foundation (AHF), World Heart Federation (WHF), International Obesity Taskforce (IOTF), International Society of Hypertension (ISH), Global Alliance for Chronic Diseases (GACD), Global Diabetes Coalition (GDC), and the Centres for Disease Control and Prevention (CDC).

In addition, the UAE-based institutional repositories searched included United Arab Emirates University, University of Sharjah, New York University, Khalifa University, and Gulf Medical University. These sources, including theses and dissertations, were searched for documents reporting on recommendations, reports, guidelines, and policies regarding HQM of the respective NCDs. Although guidelines and recommendations may constitute secondary sources synthesised from primary data, they were included to comprehensively map the quality management practises that are implemented in real-world healthcare systems.

### 2.2 Eligibility criteria

Records were included if they (1) reported on health management practises of CVD, diabetes, or obesity in the UAE context, (2) were published between 1 January 2015 and 1 March 2025, to capture recent data on healthcare practises, and (3) were available in English or Arabic.

Exclusion criteria included the following: inclusion of NCDs other than CVD, diabetes, and obesity; absence of UAE-specific data; non-primary research (e.g., literature reviews, commentaries, editorials); non-health management focus; and unavailable full text. To ensure comprehensive coverage, the reference lists of excluded reviews were manually searched to identify any potentially relevant primary records that may have been missed in the initial database search.

### 2.3 Record screening, data extraction, and analysis

The records were imported into Covidence, a record management software (21), and rigorously screened against predefined eligibility criteria by at least two independent reviewers from the team (K. S., F. A., and H. O.). The screening was conducted at the title/abstract and full-text levels. Any disagreement was resolved by the senior reviewer (Z. A.). The inter-rater reliability was determined using Cohen’s kappa statistic before conflict resolution, yielding 0.77 for title/abstract screening and 0.83 for full-text screening.

The data were extracted using a standardised pre-piloted form, capturing details across three categories: (1) publication characteristics including information on date and authors; (2) study features based on regional background, design, setting, specific NCD addressed, research objective, implemented interventions, data collection methods, key outcomes measured, main findings, and reported limitations; and (3) target population characteristics such as sample size and demographic profile. To maintain rigour and consistency, the data were independently extracted by at least one reviewer from the review team (either K. S., F. A., H. O., or F. N.) and checked for completeness of necessary details and accuracy by a senior reviewer (Z. A.). The data were extracted as presented in the records, and the authors were not contacted for additional information due to the large volume of included records. When presented with similar data from two sources, such as a thesis and an article, priority was given to the more detailed source.

Data were narratively analysed to identify patterns and trends across multiple dimensions, including study design, temporal distribution (changes in records published pre- and post-COVID-19), disease focus, population demographics, geographical coverage, types of management strategies (single/multiple and pharmacological/surgical/lifestyle), and outcomes reported.

#### 2.3.1 Subset Analysis

Since interventional studies provide the most direct evidence of active health quality improvement efforts and their outcomes, a focused thematic analysis was subsequently undertaken on a subset of interventional design. Additionally, records from the grey literature were included in the subset analysis, as they would provide the essential contextual understanding of the policy and practise environment within which interventional research operates in the UAE. Data were analysed across two dimensions. The first dimension mapped the findings to the six domains of the IOM health quality framework—namely, safety, effectiveness, patient-centredness, timeliness, efficiency, and equity (15). The second dimension classified the findings according to the Donabedian model of health quality measures—namely, outcome, process, and structural measures (16).

##### 2.3.1.1 Coding of data in Subset Analysis

To systematically classify findings, the definitions of the six IOM quality domains (15) were used to guide the coding of each study and grey literature source in the first dimension. This was carried out jointly by two reviewers (Z. A. and K. S.), resolving discrepancies through consensus.

Operational definitions and coding criteria:

1. Effectiveness, defined as the delivery of services based on scientific knowledge that yields tangible improvements in health outcomes to all who could benefit and refrains from providing services to those not likely to benefit (avoiding underuse and misuse, respectively) (15); thus, all studies that reported improvements in measured clinical outcomes (e.g., biochemical markers, anthropometric, behavioural measures) and grey literature that employed evidence-based guidelines to improve patient outcomes were coded to this domain.
2. Patient-centredness, defined as providing care that is respectful of and responsive to individual patient preferences, needs, and values and ensuring that patient values guide all clinical decisions (15). Records that addressed individualised patient services, patient values and cultural health beliefs, or the psychosocial aspects of care or patient empowerment through culturally sensitive health education were classified under this domain.
3. Safety, defined as minimising harm and ensuring that interventions do not pose undue risk to patients (15). Records that addressed adverse drug effects, investigated patient safety outcomes, or provided education focused on patient safety were included under this domain. Guidelines on structural safety requirements or screening and risk assessments were also included.
4. Timeliness, defined as the system’s ability to reduce waiting times and harmful delays for both those who receive and those who give care (15). Records that provided screening services, thereby enabling early detection/timely care and preventing delays in diagnosis, were classified under this domain. Additionally, grey literature documents that improved timely access to care *via* telehealth services or set implementation timelines to meet time-critical treatment targets were included.
5. Equity, defined as the provision of care that does not vary in quality due to personal characteristics, such as sex, ethnicity, geographic location, or socioeconomic status (15). Records that addressed specific patient populations or marginalised groups were coded to the equity domain.
6. Efficiency, defined as the ability to avoid waste, including waste of equipment, supplies, ideas, and energy (15); thus, all records that highlighted saving resources, included cost evaluations, or assessed avoidable medical expenses such as hospital admissions or surgeries were coded to this domain.

In the second dimension, following the IOM-based classification, records were further coded according to the Donabedian model of health quality measures (16). Interventions or guidelines that measured or reported clinical outcomes of medical care, or that set targets for clinical outcomes, were coded as outcome measures. Structural measures were classified as records that mentioned the setting, supporting instrumentalities, and healthcare management resources, such as the number of qualified staff, adequacy of facilities, and equipment. Records that addressed the means of providing medical services, such as screening interventions or the provision of medical treatment, were classified as process measures.

Furthermore, the records included in the subset analysis were also appraised to identify relevant WHO quality-of-care indicators by quality dimension, in line with the health quality domains, utilised in the multidimensional analysis study on quality of care and patient safety in the European region (22). These indicators included standardised preventable and treatable mortality, 30-day mortality after hospital admission for acute myocardial infarction (AMI) (falling under the effectiveness dimension); average length of stay (all hospitals, days), 1-year all-cause readmission or mortality rate after discharge from ischaemic stroke, and avoidable hospital admissions—(diabetes, rate) (falling under the efficiency dimension); patients reporting a medical mistake (falling under the patient safety dimension); doctor spending enough time with patients during consultation, doctor providing easy-to-understand explanations, and doctor involving the patient in decisions about care (falling under the patient-centredness dimension); the share of households with catastrophic health spending on average and in the poorest quintile, and the share of the population with unmet need for healthcare on average and in the poorest quintile (falling under the access dimension) (22).

## 3 Results

### 3.1 Record selection

**Figure 1:**
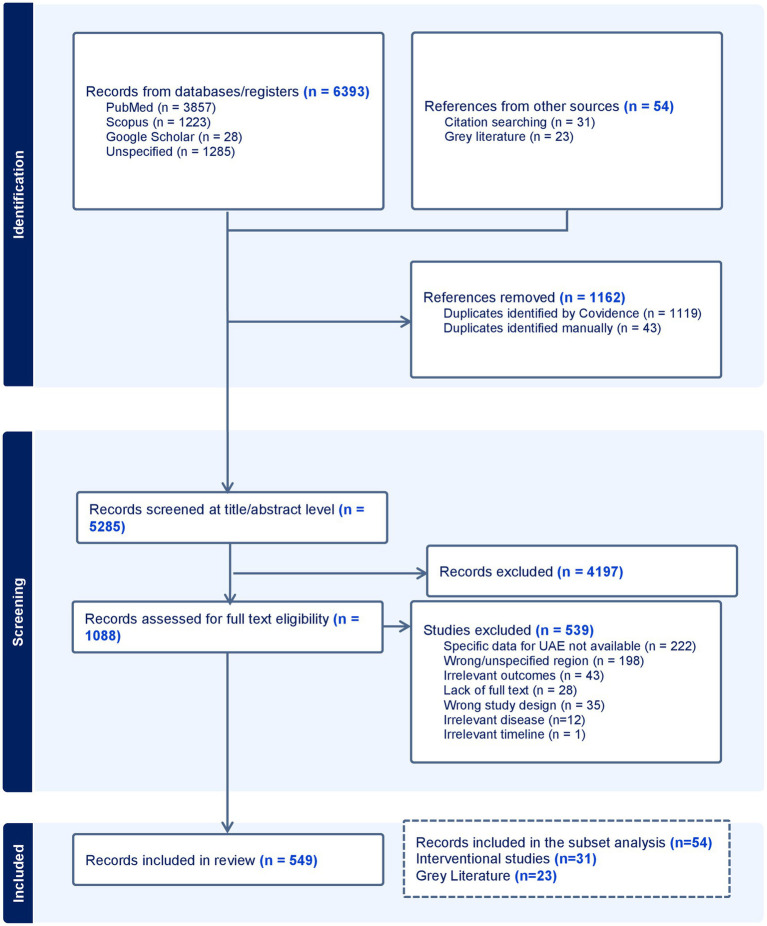
PRISMA flow chart.

A total of 6,447 records were retrieved (Figure 1), including 6,393 records from database searches and 54 from the grey literature. After removal of duplicates (*n* = 1,162), the remaining records were screened at the title and abstract level. After removal of irrelevant records, 1,088 full-text records were assessed for eligibility. Of the screened records, 539 were excluded; the reasons for exclusion were lack of UAE-specific data (*n* = 222), incorrect region (*n* = 198), irrelevant outcomes (*n* = 43), lack of full text (*n* = 28), irrelevant study design (*n* = 34), irrelevant NCD (*n* = 12), and irrelevant timeline (*n* = 1). This left us with 549 sources. For further subset analysis focusing on HQM, 54 records were included, comprising 31 interventional studies and 23 guideline/recommendation documents and reports from the grey literature.

### 3.2 Study characteristics

Temporal analysis of the total 549 records included in the scoping review revealed that 31.15% (*n* = 171) were published between 2015 and 2019, whilst the majority (68.9%, *n* = 378) emerged during or after the COVID-19 pandemic.

### 3.3 Geographical coverage

Three hundred and eight records focused on local-level data. Of these, the most represented were Abu Dhabi (*n* = 160, 51.9%), with 74 conducted in Al-Ain city, followed by Dubai (*n* = 84), Ajman (*n* = 28), Sharjah (*n* = 22), Fujairah (*n* = 6), and Ras Al Khaimah (*n* = 7). National-level records encompassing multiple Emirates numbered 143, whilst 99 records were international in scope but incorporated UAE-specific findings. These included 37 records from the broader Middle East North Africa (MENA) region, 28 from Gulf Cooperation Council (GCC) countries, and 33 with an international focus.

### 3.4 Study design

Methodologically, observational study designs were mainly reported, including cross-sectional (*n* = 283, 51.4%), cohort (*n* = 79, 14.6%), case–control (*n* = 13), and non-specified observational studies (*n* = 63, 11.5%). Intervention-based designs (*n* = 31, 5.6%) were less common, with 12 randomised controlled trials (RCTs) and 19 non-randomised intervention studies. Additional reported methodologies included qualitative research (*n* = 25, 4.6%), machine learning models (*n* = 13, 2.4%), economic and cost-effectiveness analyses (*n* = 7, 1.3%), study protocols (*n* = 3, 0.5%), and records employing multi-method approaches such as Delphi panels and questionnaire validation (*n* = 9, 1.6%). The corpus also included 23 records from the grey literature (4.2%), including 15 guideline or consensus recommendation documents, 5 reports, and 3 national or regional policy/programme reviews (addressing diabetes, diet, tobacco, and multiple NCDs).

### 3.5 Focus of records

The disease distribution showed that CVD was the most commonly addressed condition (*n* = 144, 26.2%), followed by diabetes mellitus (*n* = 133, 24.2%) and obesity (*n* = 76, 13.8%). The records also covered isolated risk factors, including smoking (*n* = 32, 5.8%), physical inactivity (*n* = 24, 4.4%), and dietary/nutritional factors (*n* = 48, 8.7%). An additional 73 records (13.3%) were dedicated to multiple NCDs, and 19 (3.5%) addressed multiple risk factors, indicating integrative research.

### 3.6 Key population groups addressed

The largest subset of records targeted individuals with diagnosed clinical conditions (*n* = 185, 33.8%), which included patients diagnosed with diabetes (*n* = 84, 15.3%), patients diagnosed with cardiovascular disease (*n* = 64, 11.7%), patients diagnosed with hypertension (*n* = 11, 2%), and patients diagnosed with obesity (*n* = 27, 4.9%). Records addressed healthy adult populations (*n* = 96, 17.5%), university students (*n* = 40, 7.3%), and children and adolescents (*n* = 58, 10.6%), including 50 addressing school-aged children and 8 addressing those aged under 6 years. Certain records also addressed specific populations involving healthcare providers (*n* = 30, 5.5%) and pregnant women (*n* = 22, 4%). Of the records providing details on participant ethnicity (*n* = 70, 12.8%), 55 included UAE nationals, whereas 15 included expatriate groups.

### 3.7 Disease management strategies

Management strategies documented in the included literature varied. A total of 280 (51%) records addressed single component management, with lifestyle interventions—primarily diet, physical activity, and behavioural change—accounting for the majority (*n* = 248, 45.2%). Pharmacological approaches were the focus of 29 records, whilst only three addressed surgical strategies in isolation (primarily bariatric or cardiac interventions). Multi-component approaches were reported in 231 (42.1%) records. Amongst these, 159 combined pharmacological and lifestyle strategies; 23 combined surgical and lifestyle; five combined surgical and pharmacological strategies; and 89 examined comprehensive tri-modality management integrating all three approaches.

### 3.8 Outcome measures reported

CVD-related outcomes included Disability-Adjusted Life Years (DALYs) (*n* = 12), Quality-Adjusted Life Years (QALYs) (*n* = 2), acute coronary syndromes (STEMI, NSTEMI) (*n* = 18), coronary artery disease (CAD) (*n* = 42), stroke prevalence, functional outcomes, anticoagulation (*n* = 32), heart failure (e.g., ejection fraction, readmissions) (*n* = 17), atrial fibrillation (*n* = 7), hypertension (*n* = 60), and dyslipidaemia (*n* = 54). Additionally, records examined healthcare delivery aspects, including cardiac diagnostics (*n* = 23), emergency care delays (*n* = 12), adherence to clinical guidelines (*n* = 10), and integration of digital health tools (*n* = 11).

Diabetes-related outcomes spanned prevalence estimates (*n* = 29), glycaemic control metrics (e.g., HbA1c, fasting glucose) (*n* = 73), medication adherence (*n* = 28), insulin therapy (*n* = 19), treatment cost assessments (*n* = 9) self-care behaviours (*n* = 17), knowledge (*n* = 12), and complication assessment (e.g., diabetic ketoacidosis, retinopathy, nephropathy, cardiovascular comorbidities) (*n* = 101).

Obesity outcomes focused on BMI trends (*n* = 107), waist circumference (*n* = 35), body composition (*n* = 22), determinants such as diet (*n* = 42), screen time (*n* = 40), sleep (*n* = 28), genetics (*n* = 5), and environmental influences (*n* = 6), as well as economic implications of obesity (*n* = 18).

### 3.9 Subset analysis

#### 3.9.1 Characteristics of interventional studies

A total of 31 interventional studies (23–53) were included in this scoping review, focusing on health management interventions targeting the three NCDs under investigation: CVD, diabetes, and obesity in the UAE context (Table 1; Supplementary Table 3).

**Table 1:** Characteristics of included interventional studies and grey literature sources.

Study characteristics		Number of studies (*n* = 31)	Number of grey literature sources (*n* = 22)
Timeline	Published before 2019 (before COVID-19)	11	5
	Published after 2019 (post-COVID-19)	20	18
Geographic distribution	Abu Dhabi	7	8
	Dubai	4	10
	Ajman	6	-
	Sharjah	2	-
	Ras Al Khaimah	2	-
	Multiple Emirates	9	5
	MENA region^1^	1	-
Study design	Randomised controlled trial	12	-
	Non-randomised interventional (pre–post study) design	19	-
	Guideline	-	10
	Programme/plan	-	3
	Recommendation	-	5
	Report	-	5
NCD addressed	Diabetes	10	4
	CVD^2^	7	4
	Obesity	1	7
	Diabetes and obesity	4	-
	Diabetes and CVD^2^	1	2
	Risk factors (e.g., diet, physical activity, smoking)	8	1
	Three NCDs^3^ and risk factors	-	5
Number of studies by participant subgroups addressed	General population	10	5
	Patients diagnosed with CVD,^2^ diabetes, and/or obesity	17	1
	Pregnant women	3	-
	Children/adolescents/university students	3	2
	Experts (healthcare professionals and policymakers)	3	15
Ethnicity of the target population	UAE^4^ nationals	5	-
	Expatriates	1	5
	General UAE^4^ population, unspecified ethnicity	25	18
Type of intervention	Pharmacological	11	-
	Non-pharmacological	15	-
	Both pharmacological and non-pharmacological	5	-
	Involved Physical Activity component	11	-
	Involved dietary intervention	11	-
	Involved in supplement use to reduce NCD^4^ risk factors	2	-
	Used technology/ digital tools	23	-
Outcomes	Reported glycaemic control	10	-
	Reported anthropometric outcomes	11	-
	Reported CVD^2^ risk factor outcomes	13	-
	Reported knowledge outcomes	13	-
	Reported change in physical activity	10	-
	Medication adherence	5	-

1 Middle East North African region.

2 Cardiovascular disease.

3 Non-communicable diseases.

4 United Arab Emirates.

Based on timeline distribution, the majority of included studies (*n* = 20, 65%) were published after 2019 (25–30, 33–45, 48, 49), whilst 11 studies (35%) were published between 2015 and 2019 (23, 24, 31, 32, 35, 46, 47, 50–53). Geographically, seven studies were from Abu Dhabi (26, 28, 32, 40, 46, 50, 52), four (23%) from Dubai (41, 42, 49, 51), six (19%) from Ajman (23, 36, 37, 45, 47, 48), two from Sharjah (31, 35), and two from Ras Al Khaimah (29, 53), whereas nine represented research from multiple Emirates within UAE (24–27, 29, 33, 38, 40, 44). One was conducted broadly across the MENA region (39) (Table 1).

Based on study design, 12 were RCTs (23–25, 27, 33, 38, 40, 41, 44, 45, 48, 49), whereas 19 followed a non-randomised interventional (pre–post study) design (26, 28–32, 34–37, 39, 42, 43, 46, 47, 50–53). Diabetes was the predominantly studied NCD for interventions (overall 10 studies), with six focusing on type 2 diabetes (23, 27, 33, 34, 42, 49), two on gestational diabetes (46, 48), one on type 1 diabetes (32), and one on both types of diabetes (25). It was followed by CVD with seven studies (37–39, 43, 45, 50, 51), and obesity with one study (20). Four studies addressed both diabetes and obesity (28, 29, 44, 47), one addressed diabetes and CVD (30). Eight studies addressed different risk factors, including diet, physical activity, and smoking (24, 30, 31, 35, 40, 41, 52, 53). In the post-COVID-19 period, a higher number of RCTs were reported to be conducted, with CVD as a more frequent focus, compared with the pre-COVID-19 emphasis on diabetes. A shift in geographic scope was also reported, with an increasing trend towards multi-emirate interventional studies, rather than the Emirate-specific approach pre-COVID-19.

A total of 4,736 participants were included in the studies, with a range of 24–439 (29, 45). Of the included studies, 17 studies were aimed at patients diagnosed with the relevant NCDs (23, 25, 27–29, 32–34, 36–38, 43–47, 50), 3 addressed pregnant women (46, 48, 53), and 10 focused on general adults whether healthy or at risk of developing the respective NCDs (24, 26, 28, 30, 35, 36, 39–42). Three studies focused on children, adolescents, and university students (26, 31, 52) and three on healthcare professionals (42, 49, 51). Regarding the ethnicity of the target population, five studies focused on UAE nationals specifically (23, 27, 28, 47, 50), whilst one study focused on expatriate school-going adolescents (52). The rest were conducted on the general population, without specific reference to ethnicity. When divided based on type of intervention, 11 interventions were pharmacological (24, 30, 33, 34, 38, 40, 42, 43, 49–51), whereas 15 studies were non-pharmacological (23, 26, 28, 31, 35, 36, 39, 41, 44, 45, 47, 48, 50, 52) and 5 had both the components involved (25, 27, 29, 37, 46). Eleven studies had physical activity component integrated in them (25–28, 31, 35, 36, 45, 47, 48, 52), whereas nine studies included dietary interventional components (25–28, 41, 46–48, 52), with two studies investigating the use of natural supplements (i.e., blackseed powder, *Acacia senegal*) to manage the NCD risk factors (24, 40). Of the included studies, 23 utilised a digital/technological component in the form of remote monitoring devices/wearables, video/multimedia, gamified learning technologies, or digital platforms, such as WhatsApp and mobile apps, for providing remote patient communication and access (23–28, 32–39, 41–47, 49, 51). The duration of interventions ranged between 1 day (single session) (51) and 5 years (46).

In terms of outcomes, 10 studies measured glycaemic control such as HbA1c or fasting glucose (23, 27, 28, 32, 34, 40, 44, 46, 48, 49), 11 studies assessed anthropometric measures, such as weight, BMI, waist circumference, and body composition (23, 24, 28, 31–35, 40, 44, 48), and 13 studies measured CVD risk factors, namely blood pressure, lipid profile, and cardiovascular function (23, 24, 28, 31, 34–38, 40, 43, 44, 50). A total of 13 studies measured knowledge change, with 4 focusing solely on knowledge outcomes (39, 42, 49, 51) and 9 focusing on knowledge as well as behavioural outcomes (26, 33, 37, 38, 41, 43, 47, 52, 53). A total of 10 studies reported changes in physical activity parameters, including engagement, levels of physical activity, and exercise capacity (23, 26–28, 31, 35, 36, 44, 46, 48). Medication adherence was assessed in five studies (23, 33, 37, 38, 43). Other outcomes included the rate of admission to hospitals for diabetic ketoacidosis (32), the rate of neonatal complications following gestational diabetes (46), and health-related quality of life measures (37).

Regarding intervention effectiveness, clear, statistically significant positive outcomes across the primary endpoints were reported in 18 studies (24, 27, 29, 31–34, 37–39, 43–46, 49–51, 53). Specific improvements were reported for HbA1c reduction (0.7–2.9%) (23, 25, 34, 48), weight loss (3.2–7.0% body weight) (27, 44, 48), blood pressure improvement (15–27 mm Hg systolic reduction) (37, 38, 43), diabetic medication adherence (23, 33, 37, 38, 50), and knowledge increase (39, 48, 49, 51, 52). Twelve studies reported mixed results, with positive outcomes for some measures and insignificant outcomes for others, or limitations affecting the outcomes (23, 25, 26, 28, 30, 35, 36, 41, 42, 47, 48, 52).

Several limitations were reported. First, methodological challenges such as limited generalisability were reported in 20 studies due to context-specific designs or narrowly defined populations (21–23, 25, 27, 29, 30, 32, 33, 35, 37–40, 42, 44–47, 49). Second, limited participant numbers leading to small sample sizes were noted in 19 studies (21–23, 26, 28, 29, 32, 33, 38, 39, 41–44, 46, 47, 50, 51). Third, short follow-up periods that limited observation of long-term effects were mentioned in 24 studies (24–31, 33–36, 38–41, 43–45, 47, 49, 51–53). Fourth, a lack of control groups or non-randomised approaches was brought up in nine studies (26, 27, 32, 33, 35, 41, 44, 45, 48). Finally, two studies noted limitations due to constraints imposed by single-site or specialised healthcare settings (21, 44).

#### 3.9.2 Characteristics of grey literature sources

A total of 23 grey literature documents were included in the study (Table 1, Supplementary Table 4) (4, 54–75). Of these, the majority were published after 2019 (4, 55–60, 62–70, 72, 75), whilst four were published before 2019 (54, 61, 71, 74). Regarding sources, 10 documents were published by DHA and pertained to Dubai; 8 pertained to Abu Dhabi and were published by DOH, Health Authority Abu Dhabi (HAAD), or ADPHC; and 4 pertained to all of the UAE and were published by MOHAP (4, 60, 72, 75). The type of documents varied between guidelines, reports, recommendations, and programmes, with four documents exclusively addressing CVD (55, 61, 69, 74), four addressing diabetes (56, 63, 67, 68), seven addressing obesity (57, 59, 60, 62, 64, 65, 70), two addressing CVD and diabetes (54, 58), and six addressing the three NCDs and risk factors (4, 66, 71–73, 75).

The target population for the guidelines and recommendations was primarily healthcare providers and policymakers, whereas two guidelines focused on the health of school children (62, 64). The reports provided national-level prevalence data and economic evaluations of the NCDs (4, 60, 72, 73, 75). Similarly, the programmes provided general information and did not specify a target population (66, 67, 71). The grey literature documents generally addressed the overall UAE population without specific reference to ethnicity. However, the National Health Survey Report and the Dubai Household Survey (2017–2018) included data on population subgroups, such as nationals, non-nationals, and migrant labourers (4, 73). Similarly, the MOHAP report on national health statistics included older adults (≥60 years) amongst the respondents (70).

Most guidelines and recommendations outlined information on diagnosis and integrated, multidisciplinary management approaches, including pharmacological and non-pharmacological management of CVD, diabetes, and obesity (54, 56, 57, 61, 63–65, 68–70). Policy documents on standards for services such as stroke care and bariatric surgery outlined structural requirements, including health facility and healthcare professional criteria for approval by health authorities (55, 59, 74). Screening programmes were described in two documents (58, 62). Amongst the plans and programmes, an NCD action plan outlined the national strategic approach and objectives to address NCDs and their risk factors (71). The remaining two programmes (66, 67) provided general information to the public about diabetes and smoking risk factors, and advocated self-efficacy in prevention and in seeking treatment. The four reports presented data on the prevalence and burden of the three NCDs in the UAE, and their associated risk factors (4, 60, 72, 73). The fifth report provided an economic case for investment to address NCDs in the UAE, detailing the direct and indirect costs of the NCD burden and the estimated costs of implementing interventions over 15 years (2020–2034) (75).

#### 3.9.3 Health quality domains

The analysis of the included records was guided by two complementary quality assessment frameworks: IOM’s quality domains (15) and the Donabedian model (structure, process, outcomes) (16), as depicted in Tables 2, 3. Additionally, they were examined for quality-of-care indicators, consistent with the multidimensional framework used in the European region study on quality of care and patient safety (22).

**Table 2:** Classification of interventional studies by the six key healthcare quality domains as defined by the Institute of Medicine and the Donabedian model of healthcare quality measures.

Number	Interventional studies	Title	Domains						Measures		
			Safety	Effectiveness	Patient-centredness	Equity	Efficiency	Timeliness	Structural	Process	Outcome
1	Abdi 2015 (23)	Behavioural lifestyle intervention study (BLIS) in patients with type 2 diabetes in the United Arab Emirates: a randomised controlled trial		✔	✔						✔
2	AlDhaheri 2017 (24)	The effect of black seed powder on blood glycaemia, blood lipidemia, and body composition on adults at risk for cardiovascular diseases		✔							✔
3	Sadiya 2016 (47)	Lifestyle intervention for weight loss: a group-based programme for Emiratis in Ajman, United Arab Emirates		✔	✔		✔			✔	✔
4	Rahmani 2016 (46)	Improving neonatal complications with a structured multidisciplinary approach to gestational diabetes mellitus management	✔	✔				✔		✔	✔
5	Deeb 2016 (32)	Implementation of a diabetes educator care model to reduce paediatric admission for diabetic ketoacidosis		✔			✔	✔	✔	✔	✔
6	Shire 2017 (51)	Stroke awareness amongst Dubai emergency medical service staff and impact of an educational intervention	✔	✔						✔	✔
7	Steen 2017 (53)	Diet and eating habits of expectant parents and families in Ras Al Khaimah, Emirates: an exploratory study			✔	✔				✔	✔
8	Al Omar 2020 (25)	The impact of a self-management educational programme coordinated through WhatsApp on diabetes control		✔	✔					✔	✔
9	Ibrahim 2022 (38)	The impact of telepharmacy on hypertension management in the United Arab Emirates	✔	✔						✔	✔
10	Shadan 2025 (49)	Diabe-teach: a randomised controlled trial of a gamified approach to enhance medical undergraduates’ knowledge and comprehension of diabetes mellitus		✔						✔	✔
11	Houjazi 2021 (37)	The impact of clinical pharmacy services on patients with hypertension		✔						✔	✔
12	Al Khatry 2023 (29)	Improvements in hepatic steatosis, obesity, and insulin resistance in adults with nonalcoholic fatty liver disease after the Primary Obesity Surgery Endoluminal 2.0 Procedure		✔							✔
13	Muthukrishnan 2021 (45)	Power walking based outpatient cardiac rehabilitation in patients with post-coronary angioplasty: randomised control trial		✔							✔
14	Mohammed 2024 (43)	Evaluation of the impact of a pharmacist-conducted hypertension clinic		✔	✔	✔				✔	✔
15	Jarrar 2021 (40)	The effect of gum arabic (*Acacia senegal*) on cardiovascular risk factors and gastrointestinal symptoms in adults at risk of metabolic syndrome: a randomised clinical trial		✔							✔
16	Jarrar 2022 (41)	Using digital platform approach to reduce salt intake in a sample of UAE population: an intervention study		✔						✔	✔
17	Mussa 2019 (44)	Personalized intervention to improve stress and sleep patterns for glycemic control and weight management in obese Emirati patients with type 2 diabetes: a randomised controlled clinical trial		✔	✔						✔
18	Dalibalta 2017 (31)	Exercise intervention on cardiovascular disease risk factors in a university population in the United Arab Emirates		✔							✔
19	Alkaabi 2021 (28)	Effects of diabetes prevention education programme for overweight and obese subjects with a family history of type 2 diabetes mellitus: a pilot study from the United Arab Emirates		✔	✔						✔
20	Hasan 2018 (35)	Counting footsteps with a pedometer to improve HMW adiponectin and metabolic syndrome amongst young female adults in the United Arab Emirates		✔							✔
21	Ali 2021 (26)	Feasibility study of a newly developed technology-mediated lifestyle intervention for overweight and obese young adults		✔	✔						✔
22	Shehab 2016 (50)	Evaluation and implementation of behavioural and educational tools that improve the patients’ intentional and unintentional non-adherence to cardiovascular medications in family medicine clinics		✔	✔						✔
23	Iskandar 2024 (39)	Evaluating the influence of a 3-min online video on the community knowledge of stroke in four Arab countries		✔						✔	✔
24	Jirjees 2024 (42)	Time for health change: promoting community-based diabetes screening and prevention with video vignettes and social marketing						✔		✔	
25	Sadiya 2022 (48)	Lifestyle intervention in early pregnancy can prevent gestational diabetes in high-risk pregnant women in the UAE: a randomised controlled trial		✔	✔						✔
26	Farooqi 2022 (34)	The impact of telemonitoring on improving glycemic and metabolic control in previously lost-to-follow-up patients with type 2 diabetes mellitus: a single-centre interventional study in the United Arab Emirates		✔				✔			✔
27	Stanley 2017 (52)	Implementation of a peer-mediated health education model in the United Arab Emirates: addressing risky behaviours amongst expatriate adolescents		✔						✔	✔
28	Ali 2024 (27)	Impact of Skills for Change Programme on metabolic control, diet, and physical activity levels in adults with type 2 diabetes: a cluster randomised trial		✔	✔				✔		✔
29	El-Deyarbi 2024 (33)	The effects of multifactorial pharmacist-led intervention protocol on medication optimisation and adherence amongst patients with type 2 diabetes: a randomised control trial	✔	✔	✔					✔	✔
30	Alzubaidi 2019 (30)	Diabetes and cardiovascular disease risk screening model in community pharmacies in a developing primary healthcare system: a feasibility study						✔		✔	
31	Hazari 2023 (36)	Effect of 8 weeks badminton session on cardiovascular and neuromuscular functions amongst older adults in United Arab Emirates: a quasi-experimental study		✔							✔

**Table 3:** Classification of grey literature sources with two-dimensional analysis, covering the six key healthcare quality domains as defined by the Institute of Medicine and the Donabedian model of healthcare quality measures.

Number	Grey literature	Source	Domains						Donabedian measures		
			Safety	Effectiveness	Patient-centredness	Equity	Efficiency	Timeliness	Structural	Process	Outcome
1	Scope of Practise and Clinical Responsibilities of Family Medicine (54)	DHA^1^		✔	✔					✔	
2	Acute Stroke Centres Inspection Checklist – Random (55)	DHA^1^	✔	✔					✔	✔	
3	EJADA^2^ Programme DIABETES KPIs^3^ and Recommendations (56)	DHA^1^		✔			✔			✔	✔
4	EJADA^2^ Programme Obesity and metabolic syndrome KPIs^3^ and Recommendations (57)	DHA^1^		✔			✔			✔	✔
5	Dubai Periodic Health Screening Guideline (58)	DHA^1^	✔	✔				✔		✔	
6	Standards for Bariatric Surgery Services (59)	DHA^1^	✔	✔	✔				✔		
7	HAAD^4^ Guidelines for the Provision of Cardiovascular Disease Management Programme (61)	HAAD^4^	✔	✔	✔				✔	✔	
8	School Screening Standard (62)	DOH^5^	✔	✔		✔		✔	✔	✔	
9	Standard for Diagnosis and Management of Diabetes Mellitus Type 1 and 2 (63)	DOH^5^	✔	✔				✔		✔	✔
10	DOH service requirements for the weight management programme for overweight and obese children (64)	DOH^5^		✔	✔	✔			✔	✔	
11	Standard for Non-surgical Management of Obesity (65)	DOH^5^	✔	✔	✔					✔	✔
12	Tobacco and Electronic Smoking Control Programme (66)	ADPHC^6^		✔	✔				✔		
13	Diabetes Prevention Programme (67)	ADPHC^6^		✔	✔					✔	
14	National NCD^7^ Action plan (71)	MOHAP^8^	✔	✔	✔	✔	✔	✔	✔	✔	✔
15	DHA^1^ Telehealth Clinical Guidelines for virtual management of Obesity (70)	DHA^1^	✔	✔	✔		✔	✔	✔	✔	
16	DHA^1^ Telehealth Clinical Guidelines for virtual management of Hypertension (69)	DHA^1^	✔	✔	✔	✔	✔	✔		✔	✔
17	DHA^1^ telehealth clinical guidelines for virtual management of Type 2 diabetes mellitus (68)	DHA^1^	✔	✔	✔	✔	✔	✔		✔	✔
18	Triage Protocol for Hyperacute Stroke Emergencies and their Referrals in Pre-Hospital and Emergency Department (ED) Setting: EMS^9^ and Self-Presenting Emergency Departments’ Arrivals (74)	DOH^5^	✔	✔	✔	✔		✔	✔	✔	
19	UAE^10^ national health survey report 2017–2018 (4)	MOHAP^8^		✔		✔	✔				✔
20	UAE^10^ National Health Survey Summary Report for Elderly Respondents (60+) (72)	MOHAP^8^	✔	✔		✔				✔	✔
21	Dubai Household Health Survey (73)	DHA^1^		✔		✔	✔				✔
22	Obesity Study, 2023 (60)	MOHAP^8^		✔							✔
23	The Case for Investment in Prevention and Control of Non-Communicable Diseases in the United Arab Emirates (75)	MOHAP^8^		✔		✔	✔	✔	✔	✔	✔

1 Dubai Health Authority.

2 EJADA refers to the United Arab Emirates’ national healthcare quality and performance measurement programme, designed to standardise indicators, benchmark providers, and drive quality improvement across the health system.

3 Key performance indicators.

4 Health Authority Abu Dhabi.

5 Department of Health—Abu Dhabi.

6 Abu Dhabi Public Health Centre.

7 Non-communicable disease.

8 Ministry of Health and Prevention.

9 Emergency Medical Services.

10 United Arab Emirates.

##### 3.9.3.1 Effectiveness

**Figure 2:**
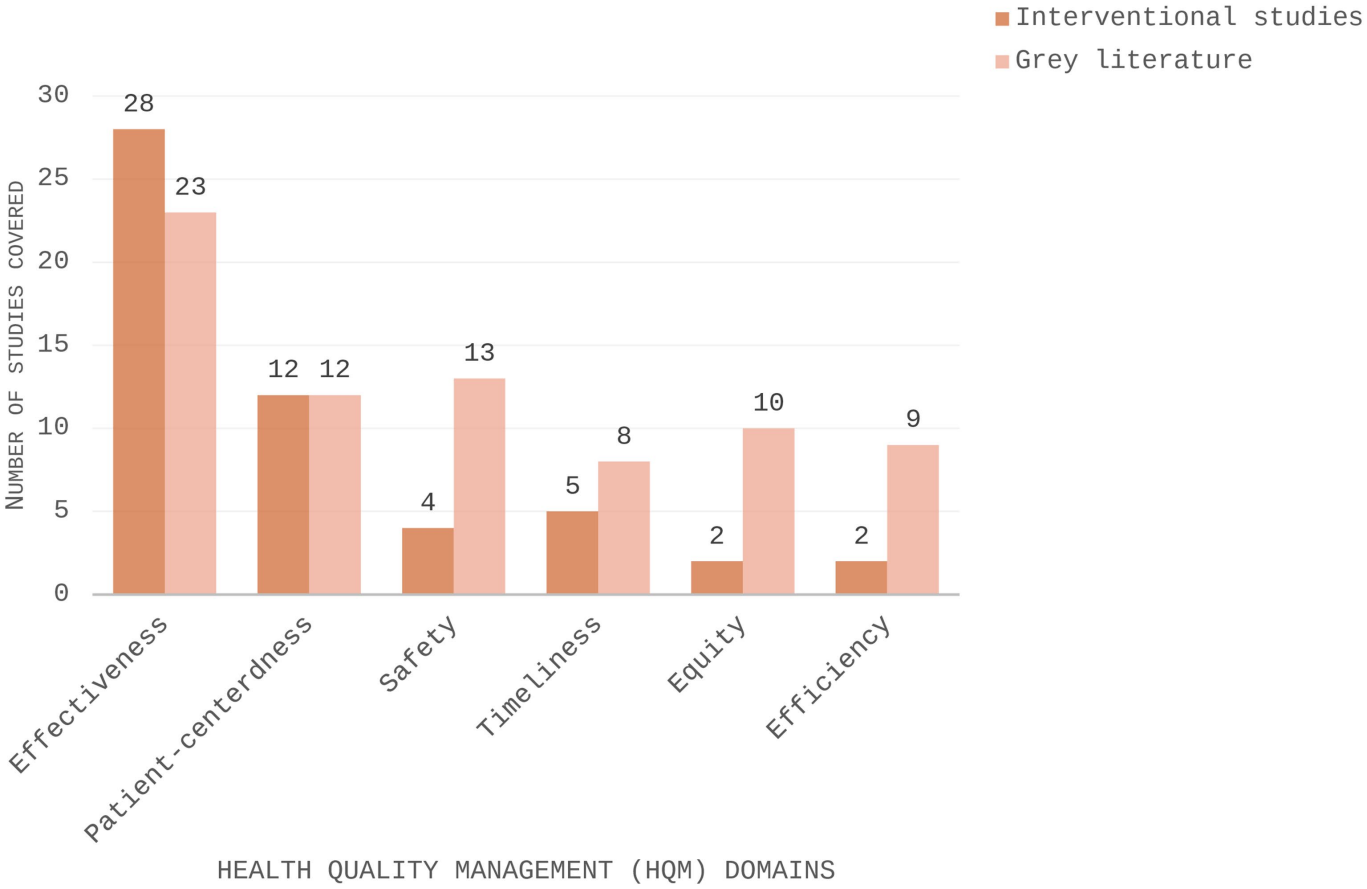
Grouped bar chart illustrating the distribution of health quality management domains in interventional studies and grey literature.

**Figure 3:**
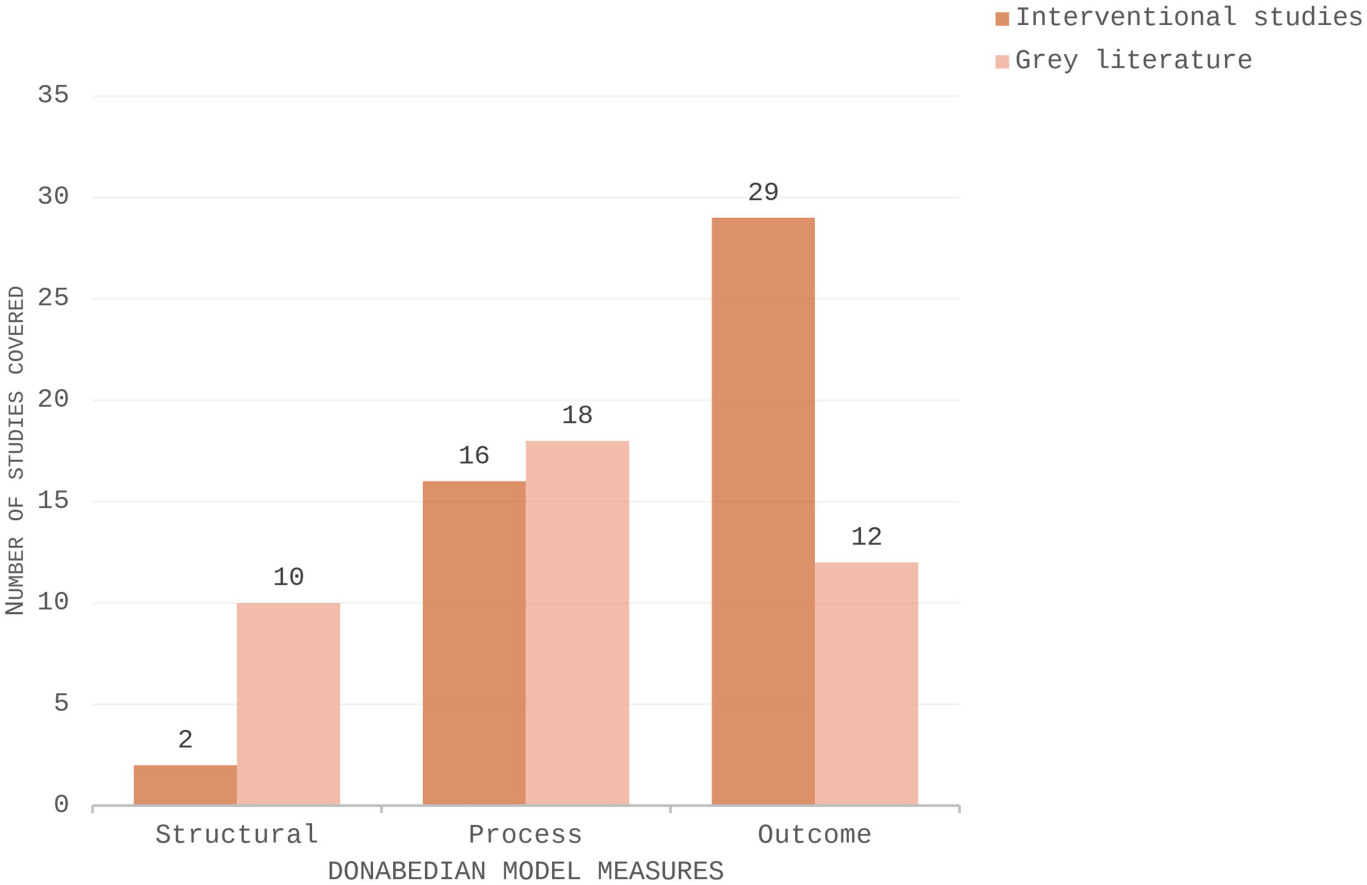
Grouped bar chart illustrating the distribution of structural, process, and outcome measures across interventional studies and grey literature.

The majority of interventional studies (*n* = 28) and grey literature documents (*n* = 23) analysed fell under this domain (Figures 2, 3). Studies reporting outcomes related to glycaemic control (*n* = 10) (23, 27, 28, 32, 34, 40, 44, 46, 48, 50); anthropometrical measures (*n* = 11) (23, 24, 26, 28, 31, 33–35, 40, 44, 48); health-related knowledge (*n* = 4) (39, 42, 49, 51); behavioural outcomes (*n* = 9) (26, 33, 37, 38, 41, 43, 47, 52, 53); CVD markers, namely blood pressure, lipid profile, or cardiovascular function (*n* = 13) (23, 24, 28, 31, 34–38, 40, 43, 44, 50), physical activity (*n* = 10) (23, 26–28, 31, 35, 36, 44, 48, 52), and medication adherence (*n* = 5) (22, 31, 35, 36, 41), were included under this domain. Grey literature documents addressing outcome measures (*n* = 12) encompassed both performance targets, such as biochemical marker thresholds or weekly exercise goals, and burden indicators, which assessed the prevalence or reduction of NCDs and associated risk factors, to guide physicians and patients in improving overall health outcomes (4, 56, 57, 60, 63, 65, 67–69, 71, 72, 75).

The interventions were primarily process-based and included approaches such as community pharmacy-based screening and support services (*n* = 4) (33, 37, 38, 43), digital patient support and health promotion services (*n* = 22) (23–28, 32–39, 41, 43–47, 49, 51), home-based health monitoring (*n* = 5) (25, 26, 34, 35, 46), disease education programmes (*n* = 5) (32, 46, 49, 51, 52), dietary programmes (*n* = 9) (25–28, 41, 46–48, 52), clinical procedures (*n* = 1) (29), supplements (*n* = 2) (24, 40), and exercise programmes (*n* = 10) (25–28, 31, 35, 36, 45, 47, 52). In total, 17 grey literature documents recommended process measures, such as service delivery and care coordination, to improve health outcomes (*n* = 17) (54–58, 61–65, 68–72, 74, 75).

Structural measures were discussed in nine studies, highlighting requirements for health system infrastructures and human resources (e.g., clinics treating specific patient groups to have certain numbers and types of specialists for respective service provision), along with governance structures and regulatory frameworks (55, 59, 61, 62, 64, 70, 71, 74, 75).

One grey literature document, the National Action Plan for NCDs (4), aligned with the WHO quality-of-care indicators for the effectiveness domain (22) by setting an objective of a 25% relative reduction in premature NCD deaths.

##### 3.9.3.2 Patient-centeredness

This domain was represented in the subset analysis with 12 interventional studies (23, 25–28, 33, 43, 44, 47, 48, 50, 53) and 12 grey literature documents (54, 59, 61, 64–71, 74) covering aspects of patient-centredness. Outcome measures were identified in studies reporting changes in weight or BMI (26–28, 47, 48), HbA1c levels (23, 25, 26, 28, 47, 48, 50), medication adherence (33, 43), and self-perceived stress (44). Grey literature documents included performance indicators related to patient education, lifestyle modification, and wellbeing (65, 68, 69, 71).

Process measures were most frequently reported. These included patient empowerment through culturally sensitive education and home-monitoring tools (23, 25–27, 43, 47, 48); provision of personalised care (23, 27, 28, 33, 43, 44, 50); and initiatives improving care coordination and communication (54, 61, 64, 65, 68–71, 74).

Structural measures were reported in fewer grey literature documents, describing policies, governance mechanisms, and human resource requirements supporting patient-centred service delivery (59, 61, 64, 70, 71, 74).

The WHO quality-of-care indicators for patient-centredness were not described in any of the documents analysed. Open data on the MOHAP website include information on complaints about private health facilities and their medical staff; however, it is non-specific (76).

##### 3.9.3.3 Safety

In total, four interventional studies explored the safety aspect of HQM, reporting both outcome and process measures (33, 38, 46, 51). Of the grey literature documents, 13 records addressed the safety domain (55, 58, 59, 62, 63, 65, 68–72, 74), of which 6 had outcome measures (63, 65, 68, 69, 71, 72), 10 had process measures (55, 58, 62–64, 68–72, 74), and 8 had structural measures included (55, 59, 61, 62, 64, 70, 71, 74). The outcome measures included neonatal safety outcomes (46), incidence of drug adverse effects identified (33, 38), healthcare worker knowledge (51), and information on the uptake of herbal remedies in older adult patients (72). On the other hand, the process measures included community pharmacists identifying and managing drug-related problems (33, 38), gestational diabetes mellitus multidisciplinary programmes (46), educational interventions for healthcare workers with a focus on patient safety in stroke detection and treatment (51), and screening programmes and guidelines addressing the safety of patients by advocating risk assessments of patients and cautioning clinicians to use appropriate therapies and avoid harmful treatments (58, 62, 63, 65). Structural measures included standards for healthcare services to meet safety requirements (55, 58, 61). No WHO quality-of-care indicators (22) were assessed in any of the documents analysed.

##### 3.9.3.4 Timeliness

Five interventional studies focused on timeliness (30, 32, 34, 42, 46). Of these, four interventional studies primarily focused on screening for NCDs, thereby enabling early detection/timely care and preventing delays in diagnosis (30, 34, 42, 46). One study reported clinical outcomes from the use of home-based self-monitoring devices, including portable glucometers and sphygmomanometers, to enable timely monitoring of NCD risk factors (34). Others focused on community pharmacy-based screening services (30, 42) and gestational diabetes screening and prevention (46), reporting process measures that evaluated how efficiently the services were delivered. Beyond NCD screening, the domain of timeliness in HQM also encompassed the prevention of disease complications, such as diabetic ketoacidosis (DKA), through timely service delivery, including health education on prevention, early recognition, and management of DKA (32). This study incorporated structural, process, and outcome measures, including educator-to-patient ratios, patient usage, evaluation of the education service, and changes in diabetic ketoacidosis hospital admission rates. Of the grey literature documents, eight addressed the domain of timeliness (58, 62, 63, 68–71, 74, 75), focusing on screening and early disease detection, the provision of care *via* telehealth services at the appropriate time, and implementation timelines with phased approaches. Each of these incorporated primarily process measures.

No WHO quality-of-care indicator under the access dimension was found to be addressed in the included records. However, open data from MOHAP show that the population with catastrophic health expenditure was 0% in 2022, addressing the access dimension (77).

##### 3.9.3.5 Equity

None of the interventional studies in this scoping review had equity as the primary focus; however, two studies did attempt to include equity as a theme by either targeting low-income communities for pharmacy-based hypertension screening programmes (43) or by targeting an underrepresented population of the less developed Emirate of Ras Al Khaimah (53). In the case of grey literature documents, equity was addressed peripherally in 10 documents by including specific population groups such as children, migrant labourers, and older adults. In total, 9 documents reported on outcome measures (11, 43, 53, 68, 69, 71–73, 75), 10 on process measures (43, 53, 62, 64, 68, 69, 71, 72, 74, 75), and 5 on structural measures (62, 64, 71, 74, 75).

None of the documents in the subset analysis assessed the WHO quality-of-care indicators (22).

##### 3.9.3.6 Efficiency

Amongst the 31 interventional studies analysed, 2 addressed this domain. Deeb et al. (2016) (32) reported a reduction in diabetic ketoacidosis admissions following a diabetes education programme, using outcome, process, and structural measures, whilst Sadiya et al. (2016) (47) evaluated the cost-efficiency of a lifestyle-based weight-loss intervention using outcome and process measures. In the grey literature, nine documents focused on outcome measures assessing avoidable hospital admissions, the costs of bariatric surgery, savings from telehealth and triage, and health expenditure trends (11, 56, 57, 68, 69, 71–73, 75). Process measures were identified in eight documents (56, 57, 68–72, 75) and structural measures in three (70, 71, 75).

Two interventional studies also aligned with the WHO quality-of-care indicators for efficiency (22). Rahmani et al. (46) reported a 2.7% increase in NICU admissions following a gestational diabetes intervention, indicating reduced efficiency, whereas Deeb et al. (32) documented a decline in DKA-related admissions from 210 to 1.8% (*p* < 0.001). Open data from the MOHAP website provide total hospital stay durations by emirate, but no data on average stay per patient are given (78). The EJADA Programme Diabetes KPIs and Recommendations (56) and the HAAD Guidelines for Cardiovascular Disease Management Programmes (61) both advocate reducing hospital admissions, though no baseline data are reported.

## 4 Discussion

This scoping review represents the first comprehensive mapping of HQM practises for CVD, diabetes, and obesity in the UAE using internationally recognised frameworks (the IOM’s Health Quality Framework and Donabedian’s model). Analysis of 549 records published since 2015 revealed a predominance of diabetes research and a rapid increase in general NCD research activity; however, most studies were cross-sectional, reflecting the descriptive emphasis typical of the existing evidence base. Subset analysis of interventional studies highlights a developing research landscape with varied representation across the six quality domains, with a heavy focus on effectiveness, moderate attention given to patient-centeredness and safety, and limited reporting on timeliness, equity, and efficiency.

Temporal analysis revealed that the post-COVID-19 period showed the adoption of rigorous study methodology, with more RCTs conducted. More studies post-COVID-19 focused on CVD than did those pre-COVID-19 on diabetes. A shift in geographic scope was also evident, with an increasing trend towards multi-emirate interventional studies rather than the Emirate-specific approach pre-COVID-19.

A greater focus on diabetes research, evident in UAE intervention studies, aligns with a broader regional pattern observed across Arab countries (79); however, this concentration highlights potential opportunities to diversify resource allocation and research priorities. Identification of facilitators, including longer intervention duration, cultural adaptability to the UAE context, multi-component approaches combining education with clinical support, involvement of specialised healthcare providers, such as diabetes educators and clinical pharmacists, and inclusion of family support, offers a roadmap for future intervention design (80). Similarly, the reported barriers in the respective intervention studies, such as difficulties in participant recruitment and retention, high dropout rates, and limited resources to sustain longer follow-up, highlight contextual factors relevant to implementation in both controlled and real-world settings (81). The substantial integration of digital health components across 23 interventional studies reflects the UAE’s strategic emphasis on healthcare innovation and digital transformation. Digital health tools and telemedicine interventions were present in eight studies, but their long-term scalability and user experience remain under-addressed in the included evidence. A systematic review and meta-analysis of mobile technology’s use to promote an active lifestyle across the broader MENA region indicates that it has a positive effect, yet reporting on user engagement is limited (82). It is noteworthy that three guideline documents for the telehealth-based delivery of the management of the three NCDs have been developed. However, assessing the extent of implementation across healthcare facilities, adherence amongst providers, and the impact on clinical outcomes, patient satisfaction, and access to care would be beneficial. The inclusion of qualified health professionals, such as pharmacists and emergency medicine staff, as well as family caregivers, in the delivery of interventions contributed to overall effectiveness. Another study in Bahrain focused on developing a culturally tailored, evidence-based, nurse-led, family-based (NLFB) intervention, highlighting the importance of empowering nurses and engaging family support systems to improve the effectiveness of diabetes management (83).

Most studies identified by the search did not directly examine the HQM of the target NCDs. Instead, they reported patient and professional perspectives on prevalence and management of these NCDs, indicating that direct examination of HQM remains limited within the available evidence. To address this, the subset analysis in this review applied an HQM framework to interpret findings and identify the gaps in current practise.

The majority of interventional studies and documents retrieved from the grey literature focused primarily on the effectiveness domain, reflecting a strong emphasis on clinical outcomes. Although this focus is aligned with international priorities for demonstrating measurable health improvements, it reflects a concentration of research activity within certain HQM domains (84). Furthermore, although policy documents, recommendations, and clinical guidelines exist, evaluation of their implementation and outcomes in real-world settings have been variably reported. This presents a challenge in determining whether the intended improvements in healthcare quality and patient outcomes are being achieved, as refinement of policy fundamentally depends on outcome evaluations (85). In the equity domain, the increased representation of different age and socioeconomic groups in national and local reports is optimistic. Yet, the underrepresentation of certain population subgroups, including migrant labourers, low-income groups, and older adults, was noted explicitly in interventional research. In several studies, age criteria in population descriptions are lacking, and there is limited research on participants younger than 18 years and older than 65 years. Geographic differences and patterns in publication were observed, with greater representation from urban Emirates than rural areas.

The relative underrepresentation of the equity and efficiency domains across both research and policy documents reflects broader systemic and institutional dynamics. National and organisational performance frameworks tend to privilege domains such as effectiveness and safety, which generate quantifiable, audit-friendly outcomes and align with accreditation standards set by the Joint Commission International (JCI) and the International Organisation for Standardisation (ISO) (86, 87). By contrast, equity and efficiency require cross-sectoral data, longer time horizons, and complex value judgements, making them more difficult to standardise or benchmark. Policy and data barriers further limit progress, as information on social determinants, vulnerable populations, and healthcare costs is often fragmented or sensitive (88). In addition, institutional incentives reinforce this imbalance—ministries and healthcare providers are typically rewarded for short-term improvements in clinical indicators or accreditation metrics, rather than for reducing inequities or improving resource efficiency (89). Addressing these gaps will require stronger integration of socioeconomic and cost-effectiveness indicators into national quality frameworks, supported by transparent data systems and intersectoral collaboration to capture the full spectrum of healthcare performance, including the wider determinants of health.

The application of the Donabedian Model further illuminates these gaps: Although process measures are commonly addressed across interventions and grey literature documents, structural and outcome measures were less frequently reported, suggesting that system-level factors such as capacity and organisational readiness for intervention implementation were not commonly explored in the available evidence.

In the subset analysis, the WHO quality-of-care indicators (22) were not commonly reported across all domains, limiting opportunities to benchmark system performance and monitor progress in NCD management within this dataset. Global initiatives such as the WHO *Global Patient Safety Action Plan 2021–2030* and the OECD’s *Health at a Glance* series emphasise the importance of harmonised indicators to track outcomes and strengthen accountability (90, 91). However, none of the UAE-based studies or policy documents analysed in this review applied the WHO quality-of-care indicators—such as patient-reported outcomes, complication rates, or access and resource-use measures (22)—to assess healthcare quality. Evidence also shows that embedding patient perspectives within routine quality assessment and service planning can improve treatment outcomes, patient satisfaction, and provider productivity (92). Similarly, incorporating economic evaluation, including cost-effectiveness analysis, can help identify affordable, high-impact interventions with the potential to substantially reduce disease burden (93). Although these findings should be interpreted within the limitations of this review, which focused on interventional studies and publicly available grey literature, they highlight an opportunity to strengthen national monitoring systems by integrating the WHO quality-of-care indicators to promote more systematic evaluation, accountability, and continuous improvement in NCD care quality.

### 4.1 Limitations

A key limitation of this review lies in the difficulty of comprehensively mapping the scope of quality management in healthcare, which would require access to internal records and outcome data from health facilities and government agencies—such as audit reports, performance metrics, and patient outcome evaluations. These documents are typically confidential and not publicly available.

In keeping with the scoping review methodology, the breadth of the research question and the inclusion of diverse evidence types led to studies varying widely in design, outcomes, and follow-up periods. This heterogeneity is inherent to scoping reviews and limits the extent to which findings can be synthesised or interpreted comparatively. Furthermore, the search strategy restricted the studies and grey literature included to those published after 2015 and to documents in English or Arabic only, thereby limiting the breadth of knowledge available. Consistent with scoping review methodology, we did not conduct a formal quality assessment, which may affect the strength of the conclusions drawn. Finally, the coding process used for two-dimensional analysis of the data may have had an element of subjectivity.

Nonetheless, this review establishes an essential foundation for understanding how HQM is conceptualised and implemented in the available literature. Future work should build on these findings through continued collaboration with government entities and healthcare providers.

### 4.2 Future implications and relevance

Future research should prioritise linking public and institutional data, enabling integration of outcome-based indicators and operational metrics *via* stronger intersectoral collaboration. Additionally, adopting a multidimensional approach to NCD quality assessment that extends beyond clinical effectiveness should be considered. Furthermore, longitudinal studies with larger sample sizes and prolonged follow-up periods are required to document the sustainability of interventions. The use of standard methodologies to compare effectiveness research would support regional benchmarking across the GCC, thereby accelerating improvements in HQM for NCDs.

The UAE’s rapidly developing health system, diverse expatriate-majority population, and mixed federal–emirate governance structure resemble many health systems undergoing fast demographic and system transitions (93, 94). By applying established HQM frameworks to the UAE evidence base, this review demonstrates how these models can be used to organise and interpret quality-related data even in settings where formal HQM structures are still emerging. These insights are relevant to other countries with similar population dynamics and evolving health system arrangements seeking to strengthen their approach to healthcare quality.

## 5 Conclusion

To the best of our knowledge, this is the first comprehensive scoping review of this kind in the UAE, mapping HQM practises for CVD, diabetes, and obesity, guided by the IOM quality domains and Donabedian model. Across 549 records published since 2015, diabetes-related research predominated, alongside a notable increase in overall NCD research activity. Interventional studies and grey literature sources demonstrated varied representation across the six quality domains in the publicly available literature, with effectiveness emerging as the dominant focus and equity as the least represented. Outcome measures were less frequently reported than process measures, reflecting a broader gap in access to performance data. By synthesising diverse evidence, this review establishes a baseline understanding of how HQM concepts are reflected in UAE NCD research and policy documents, clarifying which domains are more commonly reported and how research patterns have evolved. These findings may offer a structured overview that can support future research and deepen understanding of HQM within the UAE health system.
