# Supplementary material for: Health quality management practises for cardiovascular diseases, diabetes, and obesity care in the UAE: a scoping review
Source: Front Public Health. 2026 Jan 12;13:1703376. doi: 10.3389/fpubh.2025.1703376 (PMC12832995; doi:10.3389/fpubh.2025.1703376)
Supplement: Supplementary file 1 [file Table_1.docx]

**Supplementary Table 1: Search strategy used in PubMed**

| **Search** | **Query** | **Results** |
| --- | --- | --- |
| #7 | Search: (((Health[Title/Abstract] OR wellness[Title/Abstract] OR disease[Title/Abstract] OR well being[Title/Abstract] OR illness[Title/Abstract] OR healthcare[Title/Abstract] OR "community health"[Title/Abstract]) AND (Manag*[Title/Abstract] OR prevent* [Title/Abstract] OR promot*[Title/Abstract] OR "best practices" [Title/Abstract] OR "patient care"[Title/Abstract] OR interven* [Title/Abstract] OR "care coordination"[Title/Abstract] OR "medication adherence"[Title/Abstract] OR "risk assessment" [Title/Abstract] OR screen*[Title/Abstract] OR detect*[Title/Abstract] OR monitor*[Title/Abstract] OR recommend*[Title/Abstract] OR plan*[Title/Abstract] OR implement*[Title/Abstract] OR advoca* [Title/Abstract] OR evaluat*[Title/Abstract] OR polic*[Title/Abstract] OR "Risk factor"[Title/Abstract] OR risk[Title/Abstract] OR administer*[Title/Abstract] OR regulat*[Title/Abstract] OR control[Title/Abstract] OR strateg*[Title/Abstract] OR service* [Title/Abstract] OR system*[Title/Abstract] OR operat* [Title/Abstract] OR treat*[Title/Abstract] OR curative[Title/Abstract] OR palliative[Title/Abstract] OR rehabilitat*[Title/Abstract] OR quality[Title/Abstract] OR audit[Title/Abstract] OR organis* [Title/Abstract] OR design*[Title/Abstract] OR deliver* [Title/Abstract] OR personalis*[Title/Abstract] OR develop* [Title/Abstract] OR govern*[Title/Abstract] OR leader*[Title/Abstract] OR determin*[Title/Abstract] OR surveillance[Title/Abstract] OR equity[Title/Abstract] OR engage*[Title/Abstract] OR | 3854 |
| #6 | Search: ((Health[Title/Abstract] OR wellness[Title/Abstract] OR disease[Title/Abstract] OR well being[Title/Abstract] OR illness[Title/Abstract] OR healthcare[Title/Abstract] OR "community health"[Title/Abstract]) AND (Manag*[Title/Abstract] OR prevent* [Title/Abstract] OR promot*[Title/Abstract] OR "best practices" [Title/Abstract] OR "patient care"[Title/Abstract] OR interven* [Title/Abstract] OR "care coordination"[Title/Abstract] OR "medication adherence"[Title/Abstract] OR "risk assessment" [Title/Abstract] OR screen*[Title/Abstract] OR detect*[Title/Abstract] OR monitor*[Title/Abstract] OR recommend*[Title/Abstract] OR plan*[Title/Abstract] OR implement*[Title/Abstract] OR advoca* [Title/Abstract] OR evaluat*[Title/Abstract] OR polic*[Title/Abstract] OR "Risk factor"[Title/Abstract] OR risk[Title/Abstract] OR administer*[Title/Abstract] OR regulat*[Title/Abstract] OR control[Title/Abstract] OR strateg*[Title/Abstract] OR service* [Title/Abstract] OR system*[Title/Abstract] OR operat* [Title/Abstract] OR treat*[Title/Abstract] OR curative[Title/Abstract] OR palliative[Title/Abstract] OR rehabilitat*[Title/Abstract] OR quality[Title/Abstract] OR audit[Title/Abstract] OR organis* [Title/Abstract] OR design*[Title/Abstract] OR deliver* [Title/Abstract] OR personalis*[Title/Abstract] OR develop* [Title/Abstract] OR govern*[Title/Abstract] OR leader*[Title/Abstract] OR determin*[Title/Abstract] OR surveillance[Title/Abstract] OR equity[Title/Abstract] OR engage*[Title/Abstract] OR workforce[Title/Abstract] OR capacit*[Title/Abstract] OR financ* [Title/Abstract] OR collaborat*OR decision*[Title/Abstract] OR therap*[Title/Abstract])) AND ((((("hypertension"[MeSH Terms] OR "hypertens*"[Title/Abstract] OR "atherosclerosis"[MeSH Terms] OR "peripheral arterial disease"[MeSH Terms] OR "Cardiovascular" [Title/Abstract] OR "heart"[Title/Abstract] OR "cardiac" [Title/Abstract] OR "CVD"[Title/Abstract] OR "coronary artery disease"[Title/Abstract] OR "stroke"[Title/Abstract] OR "arrythmia*" [Title/Abstract] OR "diabetes mellitus"[MeSH Terms] OR "diabetes mellitus, type 2"[MeSH Terms] OR "hypergylcemia"[Title/Abstract] OR "overweight"[MeSH Terms] OR "obes*"[Title/Abstract] OR "noncommunicable"[Title/Abstract] OR "chronic"[Title/Abstract]) OR (smok*[Title/Abstract])) OR (alcohol[Title/Abstract])) OR (diet[Title/Abstract])) OR (activity, physical[MeSH Terms])) | 1,847,058 |
| #5 | Search: (Health[Title/Abstract] OR wellness[Title/Abstract] OR disease[Title/Abstract] OR well being[Title/Abstract] OR illness[Title/Abstract] OR healthcare[Title/Abstract] OR "community health"[Title/Abstract]) AND (Manag*[Title/Abstract] OR prevent* [Title/Abstract] OR promot*[Title/Abstract] OR "best practices" [Title/Abstract] OR "patient care"[Title/Abstract] OR interven* [Title/Abstract] OR "care coordination"[Title/Abstract] OR "medication adherence"[Title/Abstract] OR "risk assessment" [Title/Abstract] OR screen*[Title/Abstract] OR detect*[Title/Abstract] OR monitor*[Title/Abstract] OR recommend*[Title/Abstract] OR plan*[Title/Abstract] OR implement*[Title/Abstract] OR advoca* [Title/Abstract] OR evaluat*[Title/Abstract] OR polic*[Title/Abstract] OR "Risk factor"[Title/Abstract] OR risk[Title/Abstract] OR administer*[Title/Abstract] OR regulat*[Title/Abstract] OR control[Title/Abstract] OR strateg*[Title/Abstract] OR service* [Title/Abstract] OR system*[Title/Abstract] OR operat* [Title/Abstract] OR treat*[Title/Abstract] OR curative[Title/Abstract] OR palliative[Title/Abstract] OR rehabilitat*[Title/Abstract] OR quality[Title/Abstract] OR audit[Title/Abstract] OR organis* [Title/Abstract] OR design*[Title/Abstract] OR deliver* [Title/Abstract] OR personalis*[Title/Abstract] OR develop* [Title/Abstract] OR govern*[Title/Abstract] OR leader*[Title/Abstract] OR determin*[Title/Abstract] OR surveillance[Title/Abstract] OR equity[Title/Abstract] OR engage*[Title/Abstract] OR workforce[Title/Abstract] OR capacit*[Title/Abstract] OR financ* [Title/Abstract] OR collaborat*OR decision*[Title/Abstract] OR therap*[Title/Abstract]) | 6213,213 |
| #4 | Search: (((United Arab Emirates) OR (UAE)) OR (united arab emirates[MeSH Terms])) OR (Dubai OR Abu Dhabi OR Sharjah OR Ajman OR Al-Ain OR Fujairah OR "ras al khaimah" OR "Umm al Quwain") | 43,894 |
| #3 | Search: (((("hypertension"[MeSH Terms] OR "hypertens*" [Title/Abstract] OR "atherosclerosis"[MeSH Terms] OR "peripheral arterial disease"[MeSH Terms] OR "Cardiovascular"[Title/Abstract] OR "heart"[Title/Abstract] OR "cardiac"[Title/Abstract] OR "CVD" [Title/Abstract] OR "coronary artery disease"[Title/Abstract] OR "stroke"[Title/Abstract] OR "arrythmia*"[Title/Abstract] OR "diabetes mellitus"[MeSH Terms] OR "diabetes mellitus, type 2"[MeSH Terms] OR "hypergylcemia"[Title/Abstract] OR "overweight"[MeSH Terms] OR "obes*"[Title/Abstract] OR "noncommunicable"[Title/Abstract] OR "chronic"[Title/Abstract]) OR (smok*[Title/Abstract])) OR (alcohol[Title/Abstract])) OR (diet[Title/Abstract])) OR (activity, physical[MeSH Terms]) | 5537, 556 |
| #2 | Search: Manag*[Title/Abstract] OR prevent*[Title/Abstract] OR promot*[Title/Abstract] OR "best practices"[Title/Abstract] OR "patient care"[Title/Abstract] OR interven*[Title/Abstract] OR "care coordination"[Title/Abstract] OR "medication adherence" [Title/Abstract] OR "risk assessment"[Title/Abstract] OR screen* [Title/Abstract] OR detect*[Title/Abstract] OR monitor* [Title/Abstract] OR recommend*[Title/Abstract] OR plan* [Title/Abstract] OR implement*[Title/Abstract] OR advoca* [Title/Abstract] OR evaluat*[Title/Abstract] OR polic*[Title/Abstract] OR "Risk factor"[Title/Abstract] OR risk[Title/Abstract] OR administer*[Title/Abstract] OR regulat*[Title/Abstract] OR control[Title/Abstract] OR strateg*[Title/Abstract] OR service* [Title/Abstract] OR system*[Title/Abstract] OR operat* [Title/Abstract] OR treat*[Title/Abstract] OR curative[Title/Abstract] OR palliative[Title/Abstract] OR rehabilitat*[Title/Abstract] OR quality[Title/Abstract] OR audit[Title/Abstract] OR organis* [Title/Abstract] OR design*[Title/Abstract] OR deliver* [Title/Abstract] OR personalis*[Title/Abstract] OR develop* [Title/Abstract] OR govern*[Title/Abstract] OR leader*[Title/Abstract] OR determin*[Title/Abstract] OR surveillance[Title/Abstract] OR equity[Title/Abstract] OR engage*[Title/Abstract] OR workforce[Title/Abstract] OR capacit*[Title/Abstract] OR financ* [Title/Abstract] OR collaborat*OR decision*[Title/Abstract] OR therap*[Title/Abstract] | 25,987,755 |
| #1 | Health[Title/Abstract] OR wellness[Title/Abstract] OR disease[Title/Abstract] OR well being[Title/Abstract] OR illness[Title/Abstract] OR healthcare[Title/Abstract] OR "community health"[Title/Abstract] | 7,082,217 |

Supplementary table 2: Characteristics of the included interventional studies

| **Record details** | **Title** | **Region of study** | **Aim of study** | **NCD addressed** | **Study design** | **Study duration** | **Target Population and sample size** | **Method of participant recruitment/sampling** | **Details of comparator groups if present** | **Details of Intervention** | **Data collection methods** | **Key Outcomes** | **Summary of results** | **Study Limitations** |
| --- | --- | --- | --- | --- | --- | --- | --- | --- | --- | --- | --- | --- | --- | --- |
| Abdi 2015 | Behavioral Lifestyle Intervention Study (BLIS) in patients with type 2 diabetes in the United Arab Emirates: A randomized controlled trial | Ajman | To develop a behavioral lifestyle intervention program tailored to the Emirati population and evaluate its effectiveness in improving glycemic control (HbA1c levels) in patients with type 2 diabetes compared to standard care. | Diabetes Type 2 | Randomized controlled trial | The intervention lasted 6 months, with a follow-up at 1 year. | 35 Emirati nationals, aged 18–60 years, patients with diabetes and overweight | Patients referred to Rashid Center for Diabetes and Research from primary healthcare centers were assessed for eligibility and recruited during scheduled dietitian visits. | The control group received standard care, comprising three dietitian sessions at baseline, 1 month, and 6 months, with general dietary guidance and calorie-based diet plans but without behavioral strategies or structured educational materials. | The intervention group underwent a 6-month behavioral lifestyle program based on cognitive behavioral theory, including 4 individual sessions and 4 telephone calls. It emphasized SMART goal setting, self-monitoring via diet diaries, carbohydrate intake reduction, individualized weight loss targets (5–7%), and promotion of physical activity (30 minutes, 5 times a week). | Routine clinical records including HbA1c, lipid profiles, weight, body composition, blood pressure measurements, three-day food records for dietary intake, and self-reported physical activity minutes. | The primary outcome was HbA1c levels. Secondary outcomes included weight, BMI, body composition (fat mass, muscle mass, water mass), lipid profiles (LDL-C, HDL-C, total cholesterol, triglycerides), blood pressure, total and source-specific carbohydrate intake, minutes of moderate physical activity, and use of diabetes medications. | The intervention group achieved a significant reduction in HbA1c (−1.56% at 6 months, maintained at −1.12% at 1 year), whereas no significant change was observed in the control group. Carbohydrate intake, particularly from cereals, decreased significantly in the intervention group. No significant differences were found in weight, body composition, blood pressure, lipid profiles, or physical activity levels between groups. The intervention demonstrated that culturally adapted behavioral strategies can improve glycemic control among Emirati patients. | The study had a small sample size, limiting its power and generalizability. Recruitment was challenging due to appointment gaps. The study was conducted in a specialized diabetes center, which may limit its applicability to broader healthcare settings like hospitals or primary care centers. |
| AlDhaheri 2017 (Conference abstract) | THE EFFECT OF BLACK SEED POWDER ON BLOOD GLYCAEMIA, BLOOD LIPIDEMIA AND BODY COMPOSITION ON ADULTS AT RISK FOR CARDIOVASCULAR DISEASES | UAE | To measure the effect of 3 grams of black seed (Nigella Sativa) powder for 12 weeks on participants at risk for cardiovascular diseases for fasting blood glucose, HbA1c levels, blood pressure, blood lipid profile levels and waist circumference. | Risk factors for Diabetes, Obesity, CVD | Randomized controlled trial | 12 weeks | 51 Participants at risk of developing heart diseases | A controlled, randomized, single blind, parallel design study conducted on 51 participants with risk of developing heart diseases. Participants randomly assigned to consume either 3g/day of black seed powder or placebo for 12 weeks. | Participants randomly assigned to consume either 3g/day of black seed powder or placebo for 12 weeks. | Participants randomly assigned to consume either 3g/day of black seed powder or placebo for 12 weeks. | Each of weight, height, waist circumference, body composition and systolic and diastolic blood pressure were measured at baseline, mid-point (after 6 weeks) and end point (after 12 weeks). Fasting blood glucose, HbA1c, total cholesterol, LDL-C and HDL-C were tested at baseline, midpoint and end point. | Each of weight, height, waist circumference, body composition and systolic and diastolic blood pressure were measured at baseline, mid point (after 6 weeks) and end point (after 12 weeks). Fasting blood glucose, HbA1c, total cholesterol, LDL-C and HDL-C were tested at baseline, midpoint and end point. | After 12 weeks consumption of black seed powder, each of waist circumference, percent body fat, cholesterol, HDL and LDL levels were highly significantly improved (p-value < 0.01). In addition, body weight and triglyceride level were significantly improved (p-value < 0.05) | Not specified |
| Sadiya 2016 | Lifestyle Intervention for Weight Loss: a group-based program for Emiratis in Ajman, United Arab Emirates | Ajman ​ | To assess the effectiveness of the LIFE-8 program in delivering weight loss and improving glycemic control in Emiratis with obesity and/or type 2 diabetes. ​ | Obesity, Type 2 Diabetes | Non-randomized interventional study (Pre post study) | 2 years | 45 Emirati (UAE nationals) patients with obesity and diabetes type 2, aged between 18-50 years | Participants were registered through internal referrals and other healthcare facilities in the Northern Emirates. ​ Readiness was assessed using a questionnaire adapted from the American Medical Association (2003). ​ | No comparator groups were present; this was a single-arm interventional study. ​ | The LIFE-8 program was a structured, group-based lifestyle intervention combining:  Dietary Modification: Calorie-controlled diet (1,200–1,500 cal/day). ​ Partial meal replacement with Glucerna SR for 6 weeks (500 cal/day), followed by one meal replacement for the next 6 weeks (250 cal/day). ​ Group sessions on healthy eating, portion control, food labeling, and cooking.  Physical Activity: Participants were provided pedometers and encouraged to achieve 7,000–10,000 steps/day. ​ Structured exercise sessions twice a week (45 minutes each) focusing on aerobic exercises, strength training, and stretching. ​  Behavioral Therapy: ​ Self-monitoring through food diaries and pedometer activity records. ​ SMART goal setting and motivational interviewing strategies. ​ | Sociodemographic data, medical history, and weight loss history were collected using a pretested questionnaire. ​ Anthropometric measurements (height, weight, waist circumference, body composition) were taken using standardized tools (SECA electronic balance and InBody-230 bioelectric impedance instrument). ​ Glycemic control (FBG and HbA1c) was assessed. ​ Nutritional knowledge was measured using a pretested questionnaire before and after the program. ​ | Outcomes measured included  Weight Loss: ​ Average % reduction in body weight, fat mass, and waist circumference.   Glycemic Control: HbA1c and fasting blood glucose​  Sustainability: Weight loss and HbA1c was measured after 1 year  Nutritional Knowledge: paticipamts nutritional knowledge was measured and scored pre and post study.   Participant Feedback: ​ participant intervention evaluation scoring | **Weight Loss:** Participants lost an average of 5.0% body weight (≈4.8 kg) after 3 months. Fat mass reduced by 7.8%, waist circumference dropped by 4 cm. Weight loss was largely maintained (4.0%) after 1 year.  **Glycemic Control (Type 2 Diabetes):** HbA1c decreased from 7.1% to 6.6% at 3 months, and to 6.3% at 1 year. Fasting blood glucose reduced from 8.2 to 6.8 mmol/L at 3 months, and to 6.6 mmol/L at 1 year.  **Nutritional Knowledge:** Scores improved from 4/10 to 7/10 (statistically significant).  **Participant Satisfaction:** Rated 9/10 overall.  **Cost:**~$160 per participant (excluding extras), making it cost-effective for clinical use. | Small Sample Size: ​ Only 45 participants were recruited, and 28 completed the program, limiting the generalizability of the findings. Non-Randomized Design: The study was a pragmatic service assessment without a control group for comparison. ​ All participants were female, as male participants were insufficient to form a group. While weight loss was sustained for 1 year, further long-term outcomes were not assessed. ​ The program was tailored to Emirati dietary and cultural practices, which may limit its applicability to other populations. ​ Time Constraints for Male Participants: The program was conducted during morning hours on working days, which may have discouraged male participation.  The reported cost excluded meal replacements and pedometers, which are essential components of the intervention.  ​​ |
| Rahmani 2016 | Improving neonatal complications with a structured multidisciplinary approach to gestational diabetes mellitus management | Abu Dhabi | To assess the efficacy of a comprehensive and multidisciplinary gestational diabetes mellitus (GDM) program in improving neonatal outcomes. ​ | Gestational Diabetes Mellitus (GDM). ​ | Non-randomized interventional study (Pre post study) | Retrospective analysis comparing two time periods (2005–2006 and 2011–2012). ​ Study duration: One year for each time period. | 200 neonates born to mothers with GDM were included (100 neonates in each time period: 2005–2006 and 2011–2012). | Random selection of neonates born to mothers with GDM during the specified time periods. ​ | Neonates born to mothers with GDM in two distinct time periods: Group 1: 2005–2006 (before implementation of the multidisciplinary program). ​ Group 2: 2011–2012 (after implementation of the multidisciplinary program). ​ | Implementation of a structured multidisciplinary program for managing GDM. ​ Components of the program:  Screening at 24–28 weeks of pregnancy using a 75 g 2-hour OGTT. ​ Professional group diabetes education and nutritional teaching. ​ Regular follow-ups every 2–4 weeks for self-monitoring of blood glucose. ​ Insulin initiation for patients not achieving specified targets. ​ Individualized delivery plans based on metabolic control and obstetric conditions. ​ Routine non-stress tests and ultrasonography after 36 weeks to estimate fetal weight. ​ | Data collected regarding mode of delivery and neonatal complications. ​ Neonates were assessed by pediatricians immediately after delivery, and standard neonatal protocols were followed for glucose monitoring and management. ​ | Outcomes measured were changes in rates of neonatal hypoglycemia, overall neonatal complications, cesarean section rate, NICU admission rate, incidence of macrosomia and number of neonates without postnatal complications. ​ | Neonatal hypoglycemia: Significant reduction from 16.6% in 2005–2006 to 3.7% in 2011–2012 (P = 0.006). ​ Overall neonatal complications: Significant improvement, with a reduction from 30% in 2005–2006 to 16% in 2011–2012 (P = 0.04). ​ Cesarean section rate: Increased from 12% in 2005–2006 to 22% in 2011–2012 (P = 0.013). ​ NICU admissions: Increased from 3% in 2005–2006 to 5.7% in 2011–2012 (P = 0.017). ​ Macrosomia: Trend toward decreased incidence (from 7.3% in 2005–2006 to 4.5% in 2011–2012), but not statistically significant (P = 0.64). ​ Neonates without complications: 14% rise in the number of neonates without postnatal complications. ​ ​ | Sample size: The small number of cases of macrosomia may have limited the ability to detect statistically significant differences in this outcome. ​ Retrospective design: The study relied on retrospective data, which may introduce biases or limit the ability to control for confounding factors. Single-center study: Conducted at Tawam Hospital in the UAE, which may limit the generalizability of findings to other regions or healthcare settings. ​ Limited focus on maternal outcomes: The study primarily focused on neonatal outcomes, with less emphasis on maternal health outcomes. |
| Deeb 2016 | Implementation of a Diabetes Educator Care Model to Reduce Paediatric Admission for Diabetic Ketoacidosis | Abu Dhabi | To study the impact of implementing a diabetes educator care model on reducing hospital admissions for diabetic ketoacidosis (DKA) in children and adolescents | Diabetes type 1 | Non-randomized interventional study (Pre post study) | Duration: 5 years (2009–2014) | Patients under 18 years and their carers. The number of patients followed up in the outpatient diabetes clinic increased from 37 in 2009-2010 to 331 in 2013-2014.  ​ | Participants were identified through the Mafraq Hospital Health Information System (HIS) and the outpatient diabetes clinic database. | No explicit comparator groups were mentioned.  The study compared the baseline year (2009-2010) with subsequent years after implementing the diabetes educator care model. | Intervention: Implementation of a diabetes educator care model.  ​ Four nurses were trained as diabetes educators to provide structured education, 24/7 telephone access, and direct support to patients and families. Patients were allocated a named diabetes educator and provided with open access to the diabetes clinic.  ​ Education focused on DKA recognition, prevention, and management, including the use of blood and urine ketone strips.  ​ Group education meetings, awareness campaigns, and training on diabetes management devices were conducted. | Data on hospital admissions for DKA was obtained from the Mafraq Hospital Health Information System (HIS) and ward records.  ​ Quarterly data collection was conducted by a study team member, and the information was compiled in a shared folder.  ​ The outpatient diabetes clinic database was updated every 3 months to include all patients under follow-up. | The percentage of DKA admissions, ​ the ratio of admissions to outpatient follow-up patients.  ​ Secondary outcomes measured included measuring the trends in number of phonecalls received by the diabetes educators. | The diabetes educator care model led to a significant reduction in hospital admissions for diabetic ketoacidosis (DKA) in children and adolescents. DKA admission rates dropped from 210% in 2009–2010 to just 1.8% in 2013–2014, with a 210-fold reduction in the ratio of admissions to outpatient cases (from 2.1 to 0.01), which was statistically significant (p = 0.001).  Over the five-year period, there were 158 DKA admissions among children and adolescents. However, the admission rate consistently declined each year. During this time, each diabetes educator's patient load increased significantly—from about 15 patients at the start to around 90 patients by 2014. Educators also received an average of five phone calls per week from patients, with more frequent calls coming from younger children and those using insulin pumps. These calls were especially common during the early phase of pump use and during Ramadan, when fasting impacted diabetes management. | The study lacked a control group, making it difficult to attribute reductions in diabetic ketoacidosis (DKA) admissions solely to the educator care model. Other factors—such as changes in diabetes treatments, insulin regimens, or overall improvements in care—may also have contributed. The research was conducted at a single hospital in Abu Dhabi, which limits its generalizability to other regions or healthcare systems.  Resource constraints were significant. Training diabetes educators to a high level is costly, and such specialists are not widely available. Institutions faced challenges in developing unified education materials and obtaining necessary diabetes management tools like ketone-detecting strips. Additionally, many patients were not fully insured, and some insurers did not cover essential diabetes accessories, posing a barrier to comprehensive care.  Finally, the study relied on hospital and outpatient records, which may contain inaccuracies or incomplete data, further affecting the reliability of the findings. Sustaining such a care model can be difficult in settings with limited resources. |
| Shire 2017 | Stroke awareness among Dubai emergency medical service staff and impact of an educational intervention | Dubai | To assess the baseline awareness of EMS staff in Dubai regarding pre-hospital identification and management of acute stroke and to evaluate the immediate impact of an educational intervention on their knowledge. | CVD | Non-randomized interventional study (Pre post study) | Conducted over two weeks in April 2014 | 274 Emergency medical services EMS staff members. | Convenience sampling based on availability and willingness to participate | Comparison of knowledge scores before and after an educational lecture | A single interactive lecture based on AHA/ASA guidelines covering stroke symptoms, mimics, thrombolysis, and pre-hospital management | Pre- and post-intervention questionnaire covering stroke types, symptoms, mimics, pre-hospital care steps, and thrombolysis (total score: 27) | Measured outcomes included EMS staff knowledge of stroke symptoms, mimics, and thrombolysis, and pre-hospital management steps. The main outcome was the change in knowledge score before and after an educational session. Subsection scores were also analyzed. | Baseline knowledge among EMS staff was suboptimal: only 6.6% correctly identified stroke mimics, and none knew the thrombolysis window. Post-intervention, knowledge improved dramatically across all domains—stroke mimics (88.3%), therapeutic window for thrombolysis (87.2%), and pre-hospital management (from 40% to 68.2%). The mean score increased from 17.29 to 24.81 (difference: 7.53, p<0.001). EMS staff with less than 5 years’ experience performed better post-intervention. The findings suggest that a simple educational session can significantly improve EMS knowledge and may enhance pre-hospital stroke care. The baseline awareness of most aspects of acute stroke identification and management was poor in our  EMS participants. Our educational lecture proved effective in improving this knowledge when tested immediately  post intervention. However, there is a need to re-assess this at periodic intervals to identify the need for refresher  courses on pre-hospital stroke management | The study only assessed immediate knowledge retention and did not evaluate long-term impact or behavioral change in actual practice. The language of instruction (English) may have been a barrier for some EMS staff from diverse linguistic backgrounds. Additionally, convenience sampling may limit generalizability, and the study did not assess whether improved knowledge translated into improved clinical outcomes such as thrombolysis rates or reduced pre-hospital delays. |
| Steen 2017 | Diet and eating habits of expectant parents and families in Ras Al Khaimah, Emirates: An exploratory study | Ras Al Khaimah | Aim. To explore the diet and eating habits of expectant parents and their families during pregnancy and test the feasibility of introducing an EatWell Assist workshop and diary, to increase awareness of healthy eating to improve family diet and nutritional status. | Diet | Non -randomized interventional study (Pre post study) | Study Period: November 2015 – November 2016 (12 months).  Fieldwork Dates: February and May 2016. | Pregnant women and their husbands. Phase 1 (Interviews): 20 expectant mothers, 10 expectant fathers.  Phase 2 (Workshops): 15 expectant mothers, 5 female family members | Purposive sampling from three hospitals in RAK (RAK Hospital, Saqr Hospital, Sham Hospital). | No formal comparator group, but themes compared between expectant mothers and fathers. | EatWell Assist Workshop:  Covered healthy eating, portion sizes, hydration, and dispelling myths.  Included a food diary for 4 weeks post-workshop. | Data collection occurred in 2 phases:  Phase 1: Face-to-face interviews (45-60 mins, guided by an interview schedule).  Phase 2: Workshop evaluations (Likert-scale questionnaire). EatWell food diary (recorded daily intake). | Key outcomes measured included  -Knowledge gaps (portion sizes, hydration).  -Fast food reliance.  -Cultural myths (e.g., avoiding camel meat).  Workshop success was measured by assessing healthy eating awareness and changes in eating habits as seen in food diaries. | Expectant parents' diets were influenced by Western fast food and cultural habits. While workshops improved nutritional knowledge, fathers’ participation was low due to work commitments. Food diaries helped parents modify behaviors, such as increasing water intake and adjusting portion sizes.  Seven main dietary themes were identified for mothers and five for fathers, including knowledge, eating patterns, fast foods, preferences, and influences. Digital tools like the EatWell Assist workshop were commonly suggested for delivering healthy eating education.  Of 20 food diaries distributed, 12 were returned. Mothers found the diaries helpful, easy to use, and effective for tracking food intake and learning about portion sizes through clear visual guides. The diaries showed promise for supporting healthy eating among pregnant women in the UAE. | The study had several limitations. It involved a small sample size and a short follow-up period (4 weeks), limiting generalizability. No expectant fathers attended the workshops, likely due to cultural or work-related barriers. Data relied on self-reporting through food diaries, which may introduce bias.  Language barriers were a potential challenge, but mitigated through local coordinators, interpreter support, and bilingual facilitators. While exploratory in nature, the study still offered valuable insights into the dietary habits of expectant parents in the UAE and laid the groundwork for future research and expanded evaluations. |
| Omar 2020 | The impact of a self-management educational program coordinated through whatsapp on diabetes control | UAE | To assess the impact of a patient-centered diabetes education program administered through WhatsApp on glycosylated hemoglobin (HbA1c) values and to evaluate the correlation between health literacy and numeracy on intervention outcomes. | Diabetes Mellitus (Type 1 and Type 2) | Randomized controlled trial | Study start date: Approved on January 19, 2019  ​ Study duration: 6 months (intervention phase) | 164 Adult DM patients aged 18 to 80 years. | Participants were conveniently recruited from private medical centers across seven emirates in the UAE. | Intervention group: Received diabetes self-management education through WhatsApp (109 participants initially, 84 completed)  ​ Control group: Received usual care without the WhatsApp intervention (109 participants initially, 80 completed)  ​ | Intervention: Daily educational information related to diabetes self-management behaviors (AADE7 Self-Care Behaviors®) sent through WhatsApp.  ​ Content: Messages about healthy eating, food portion management, physical activity, self-monitoring of blood glucose, medication reminders, insulin use, and coping with diabetes.  ​ Procedure: WhatsApp group initiated and moderated by licensed pharmacists Messages translated to Arabic and adapted to local cultural context  ​Private bidirectional communication allowed participants to seek advice and get feedback   Meanwhile, participants in the control group were offered usual diabetes care without the WhatsApp intervention; they only received phone calls 6 months into the study to obtain their most recent HbA1c value. | Data collection tools (Details on how data was collected?):  Health literacy assessment: Literacy Assessment for Diabetes (LAD) and Diabetes Numeracy Test (DNT)  ​ HbA1c values: Collected at baseline, 3 months, and 6 months for the intervention group; baseline and 6 months for the control group  ​ Participant satisfaction: Assessed online through WhatsApp using a self-developed satisfaction tool | The main outcome of the study was the mean change in HbA1c level by comparing the control group and intervention group values at different time intervals. Secondary outcome was participant satisfaction with the intervention in terms of benefit and sustainability. | Summary of results: HbA1c Reduction: Intervention group: Significant reduction in HbA1c from 8.4% (SD 1.06) to 7.7% (SD 1.35) after 6 months (p=0.001).  ​ Control group: Minimal reduction in HbA1c from 8.5% (SD 1.29) to 8.4% (SD 1.32), not clinically significant (p=0.032).  ​ The difference in HbA1c reduction between the groups was 0.6%, which is considered clinically significant.  ​ Health Literacy and Numeracy: No significant correlation between health literacy (LAD scores) or numeracy (DNT scores) and HbA1c changes.  ​ Age and Diabetes Type: Greater HbA1c reduction in participants younger than 30 years compared to older participants.  ​ Type 2 diabetes patients showed a greater reduction (0.8%) compared to type 1 patients (0.4%).  ​ Participant Satisfaction: 80% found the WhatsApp intervention beneficial.  ​ 67% found it convenient.  ​ 90% preferred to continue using the intervention long-term.  ​ | Study Limitations: Dropouts: A significant number of participants dropped out (164 completed out of 218), which may have affected the study's power. Message Engagement: The researchers could not confirm whether all WhatsApp messages were opened and read by participants.  ​ Confidentiality: Potential issues with confidentiality if others accessed participants' WhatsApp messages.  ​ Short Duration: The intervention lasted only 6 months, limiting the ability to assess long-term outcomes.  ​ Sample Size: The effective sample size (84 in the intervention group and 80 in the control group) was smaller than initially planned, potentially compromising statistical power.  ​ Generalizability: The study was conducted in the UAE, and findings may not be generalizable to other regions or populations. |
| Ibrahim 2022 | The impact of telepharmacy on hypertension management in the United Arab Emirates | UAE | To assess the effectiveness of telepharmacy services delivered by community pharmacies in hypertension management and examine its influence on pharmacists’ ability to identify drug-related problems (DRPs). | CVD | Randomized controlled trial | Start Date: June 2021  ​ Duration: 12 months | 229 Adults with uncontrolled hypertension | Recruitment: 16 community pharmacies were purposively sampled based on their ability to provide telepharmacy or traditional pharmaceutical services. ​ Each pharmacy recruited 15 patients by screening customers against eligibility criteria. ​ Participants provided written informed consent. ​ | Intervention Group (IG): Received telepharmacy services, including virtual meetings every two weeks for six months and monthly meetings for the next six months. ​ Services included medication review, BP monitoring, dose adjustments, lifestyle counseling, and home delivery of medications. ​ Control Group (CG): Received traditional pharmaceutical services, including face-to-face consultations and BP measurements. ​ | Telepharmacy Services: Included virtual counseling, remote prescription filling, home delivery, and regular follow-ups via phone/video calls or messages. ​ Pharmacists reviewed BP readings, adjusted medications, and provided lifestyle advice. ​ | Data Collection Form: Designed in Arabic and English, filled out by pharmacists over the study period. ​ BP readings were taken at baseline, 3, 6, 9, and 12 months. ​ Medication adherence was assessed using the 8-item Morisky Medication Adherence Scale. ​ Drug-related problems (DRPs) were classified using AbuRuz et al.' ​s tool. | Key Outcomes:  Changes in blood Pressure between IG and CG at 3, 6, 9 and 12 months. ​  Medication adherence in IG compared to CG at 12 months.  improvements in knowledge of hypertension symptoms, risk factors, and healthy lifestyle behaviors. ​  Drug-Related Problems (DRPs) reported by IG and CG and pharmacist interventions related to patient education, dose adjustments, and therapy additions. ​ | Blood Pressure Reduction:  Intervention Group (IG): Systolic BP dropped from 145.9 to 124.9 mmHg; diastolic from 84.3 to 77.8 mmHg.  Control Group (CG): Systolic BP dropped from 146.7 to 132.4 mmHg; diastolic from 85.1 to 81.9 mmHg. Significant improvements in IG at 3, 6, and 9 months; 12-month systolic BP difference was not statistically significant.  Medication Adherence:  Improved more in the IG (score increase from 4.9 to 7.6) vs. CG (4.6 to 6.3).  Knowledge Gains:  IG participants showed better understanding of hypertension symptoms, risk factors, and lifestyle changes.  Drug-Related Problems (DRPs):  Higher DRP detection in IG (2.1% vs. 1.0% in CG); more DRPs per patient (0.6 vs. 0.3).  Efficacy- and safety-related DRPs were more frequently identified in IG.  Pharmacist Interventions:  More interventions in IG (331 vs. 196 in CG), especially in education, dose adjustments, and therapy additions. | Self-Reporting Bias: Data relied on pharmacists' self-reports, risking bias and reduced reliability.  Limited Outcomes: Focused on process measures (e.g., BP, DRPs, adherence), not clinical outcomes like mortality or hospitalization.  No Cost/Time Analysis: Did not assess economic or time differences between telepharmacy and traditional services.  Missing Data: The impact of missing data was not evaluated, despite the high volume of customers.  Sustainability: Long-term effectiveness may decline without ongoing tool optimization.  Lack of Collaboration Insight: Did not explore pharmacists' collaboration with other healthcare providers. Further studies are needed to optimize therapy and patient safety. |
| Shadan 2025 | Diabe-teach: a randomized controlled trial of a gamified approach to enhance medical undergraduates' knowledge and comprehension of diabetes mellitus. | Dubai | To evaluate the effectiveness of the Diabe-teach board game in enhancing diabetes knowledge retention and engagement among medical undergraduates, in comparison to traditional self-study methods. | Type 2 Diabetes Mellitus | Randomized controlled trial | Study conducted in 2022 over a two-hour single intervention session | 56 University students (medical undergraduates (Years 1–3)) | Randomized assignment using computer-generated algorithm (Randomizer.org) | Control group: Structured self-study Intervention group: Diabe-teach board game session (identical content) | Diabe-teach board game focused on diabetes pathophysiology, clinical features, investigations, and management using keyword-based interactive gameplay | Pre- and post-test with 10 MCQs; anonymous feedback survey (Likert-scale) on the gamified experience | The study measured knowledge retention on diabetes through changes in pre- and post-test scores, and evaluated student engagement, communication, confidence, and self-awareness of knowledge gaps via feedback survey. The distinction between correct, incorrect, and "I don't know" answers was also used to assess critical self-assessment and metacognition. | Both groups showed significant knowledge improvement, but the Diabe-teach group outperformed the control group (mean post-test score: 81.8% vs. 68.9%, p < .001). The intervention group also showed a higher frequency of “I don’t know” responses, indicating better self-awareness and reduced guessing. Feedback revealed 100% of students preferred the game over self-study and reported improved confidence, peer communication, and deeper understanding. The game was considered easy to use and enjoyable, and participants unanimously supported its future integration into medical education. The findings suggest  that the “Diabe-teach” board game is an effective tool for teaching complex medical concepts, fostering both knowl- edge retention and critical self-assessment among students. These results contribute to the growing body of literature  on game-based learning, supporting its potential as a valuable adjunct to traditional medical education methods.  Further research could explore the application of this approach across other medical disciplines. | The study had a small sample size from a single institution, limiting generalizability. The evaluation focused on short-term knowledge retention without long-term follow-up. Self-reported feedback could introduce bias, and potential confounders such as academic performance were not controlled for. Future studies should assess longitudinal effects and explore broader applications across disciplines. |
| Houjazi 2021 | The impact of clinical pharmacy services on patients with hypertension. | Ajman | To evaluate the impact of clinical pharmacist interventions on blood pressure, medication adherence, and quality of life in hypertensive patients in outpatient settings in the UAE. | CVD | Non-randomized interventional study (Pre post study) | Study conducted between August 1, 2019, and July 31, 2020 (12 months) | 160 Hypertension Patients aged 18–60, | Convenience sampling from cardiology and internal medicine clinics at Thumbay Hospital and Clinic |  | Clinical pharmacists provided three medication counselling sessions over 12 months (initial in-person, followed by phone or in-person at 4- and 8-month intervals). Sessions covered medication education, adherence, lifestyle changes, and side effect reporting. | ALPK2 sphygmomanometer for SBP and DBP  MINICHAL (Spanish Hypertension Quality of Life questionnaire)  DAI-10 (Drug Attitude Inventory for adherence) | Key outcomes measured were changes in SBP, DBP, Quality of life (MINICHAL score), Medication adherence. | The study demonstrated that clinical pharmacy services, particularly structured counselling sessions, significantly improved blood pressure control, medication adherence, and quality of life among newly diagnosed hypertensive patients in the UAE. The SBP and DBP reductions exceeded those reported in several comparable international studies, and the majority of participants shifted from nonadherent to adherent status. This underscores the vital role of pharmacists in chronic disease management, particularly in regions like the Gulf where such services are underutilized. | No control group; quasi-experimental design  Limited generalizability due to single-center setting and sample characteristics  Self-reported adherence may be subject to bias  Only three counselling sessions provided; more frequent engagement could yield greater impact |
| AlKhatry 2023 | Improvements in hepatic steatosis, obesity, and insulin resistance in adults with nonalcoholic fatty liver disease after the primary obesity surgery endoluminal 2.0 procedure. | Ras al Khaimah | To evaluate the effectiveness and safety of the POSE 2.0 procedure in improving hepatic steatosis, obesity, and insulin resistance among adults with NAFLD. | obesity, Type 2 diabetes | Non-randomized interventional study | Approved in January 2020; conducted from January 2020 to March 2021 | 42 Adult patients with obesity (BMI ≥30) and liver disease | Self-allocation based on treatment preference to reflect real-world clinical practice and improve engagement and retention. | POSE 2.0 + lifestyle modification vs. lifestyle modification alone | POSE 2.0 involves endoscopic full-thickness plications of the stomach to reduce gastric volume. Both arms received high-intensity lifestyle modification counseling (caloric restriction, physical activity, coaching). | FibroScan (CAP and liver stiffness)  Blood tests: liver enzymes, glucose, insulin, lipids  Anthropometry  Patient-reported outcomes (Three-Factor Eating Questionnaire 18, Gastroparesis Cardinal Symptom Index) | Key outcomes measured were resolution of hepatic steatosis between POSE 2.0 arm and control group by measuring CAP Controlled Attenuation Parameter — a non-invasive ultrasound-based measurement), ALT, AST, HIS and APRI, %TBWL, HbA1c, insulin, and postprandial glucose.  Serious adverse events were alos assessed. | POSE 2.0 led to significant and sustained reductions in hepatic steatosis, obesity, and insulin resistance over 12 months compared to lifestyle modification alone. Over half of the POSE 2.0 group achieved complete resolution of steatosis, and nearly 75% reached ≥10% TBWL — a threshold associated with histologic improvement in NAFLD. Biochemical markers of liver health (ALT, AST, HSI, APRI) and insulin resistance improved substantially in the POSE 2.0 group. This study provides real-world, prospective evidence supporting the efficacy and safety of endoscopic bariatric interventions in a UAE population. | No histologic confirmation (no liver biopsies)  Small, non-randomized, single-center sample  Unmeasured patient engagement/compliance  Most participants had early-stage fibrosis at baseline  Generalizability to non-obese NAFLD patients is unclear |
| Muthukrishnan2021 | Power walking based outpatient cardiac rehabilitation in patients with post-coronary angioplasty: Randomized control trial. | Ajman | To assess whether adding treadmill-based power walking to standardized outpatient cardiac rehabilitation (SOCRP) improves health-related quality of life (HQoL), functional exercise capacity (FEC), left ventricular ejection fraction (LVEF), and metabolic equivalent of tasks (METs) in patients following coronary angioplasty (CA). | CVD | Randomized controlled trial | Conducted between April and September 2020; intervention lasted 4 weeks | 24 Adult CVD patients aged 30–70 | Recruited from cardiac units and medical records at Thumbay Hospital Dubai; block randomized | Intervention: SOCRP + treadmill-based power walking  Control: SOCRP only (including graded treadmill walking) | Both groups: 12 sessions over 4 weeks (3/week) including aerobic, strength, and flexibility exercises  Intervention group received progressively intense power walking guided by heart rate zones  All participants tracked daily steps using a pedometer app | 6-minute walk test (6MWT)  Echocardiogram (LVEF)  Exercise stress test (METs)  HeartQoL questionnaire  Smartphone pedometer app (StepUp) | Compared to the control group, the intervention group showed significantly greater improvements:  HQoL (Global score): +155% vs. +75%  6MWT Distance: +24% (95.9m) vs. +9% (37.5m)  LVEF: +13% (7.34% absolute) vs. +4.8% (2.75%)  METs: +35% vs. +13.5%  Steps: Intervention group averaged 8,632 steps/day vs. 5,003 in control Positive correlations found between step count and HQoL, METs, and FEC | The study concluded that incorporating treadmill-based power walking into a standard cardiac rehabilitation program significantly improves functional capacity, ejection fraction, aerobic fitness, and quality of life in post-angioplasty patients over a short 4-week period. Patients in the intervention group were more active, walked further, and reached higher METs. The pedometer feedback likely contributed to better motivation and exercise adherence. This model offers a scalable, low-cost enhancement to outpatient cardiac rehab in similar settings. | Small sample size (n=24)  Single-center study  Short duration (4 weeks)  Pandemic-related constraints may have influenced participation and outcomes  Results not generalizable to all cardiac patients, especially those with reduced ejection fraction |
| Mohammed 2024 | Evaluation of the Impact of a Pharmacist-Conducted Hypertension Clinic. | DUBAI | To evaluate the impact of a pharmacist-led hypertension clinic on blood pressure control and patient satisfaction in a community pharmacy serving a low-income population. | CVD | Non-randomized interventional study (Pre post study) | Study conducted over 13 months, beginning April 2022 | 30 Adult patients with hypertension aged 18–65 yrs | Population-based, all eligible patients from the pharmacy were enrolled consecutively | Not applicable — single group study | Pharmacist-led hypertension clinic providing:  BP monitoring (monthly)  Medication verification  Lifestyle and dietary counseling  Referral to low-cost physicians for tests (lipids, renal, liver)  Personalized follow-up and education during pharmacy visits (~30 min/session) | BP readings via validated equipment (as per ACCP/ASHP 2022 guidelines)  Google Forms for data entry by pharmacist  Patient satisfaction survey (adapted from validated PROMIS and pharmacist-led service tools) | ➤ Primary Outcomes:  Change in systolic and diastolic BP over time  Patient satisfaction with pharmacist-led services  ➤ Secondary Outcomes:  Lifestyle change adherence  Medication usage patterns  Willingness to pay for pharmacist services | Systolic BP decreased significantly from 155 mmHg to 128 mmHg (mean difference: –27 mmHg; P = 0.001)  Diastolic BP decreased from 95 mmHg to 82 mmHg (not statistically significant; P = 0.17)  8 patients (26.6%) managed BP with lifestyle changes alone (no medication)  Most participants were overweight or obese  100% male, mean age ~43 years, 83.3% reported physical activity  High patient satisfaction:  100% agreed that pharmacist counseling improved care  73.3% were willing to pay out-of-pocket for continued services  Frequent follow-ups: Most patients visited monthly for 6–13 months  Survey Cronbach’s alpha = 0.895 (high internal consistency) | Small sample size and lack of control group  Conducted in one pharmacy serving low-income men — not generalizable to all UAE populations  Self-reported satisfaction may be influenced by Hawthorne effect  No assessment of long-term medication adherence or complication prevention  Collaborative care with physicians was limited by setting |
| Jarrar 2021 | The Effect of Gum Arabic (Acacia Senegal) on Cardiovascular Risk Factors and Gastrointestinal Symptoms in Adults at Risk of Metabolic Syndrome: A Randomized Clinical Trial. | Abu Dhabi | To investigate the effects of daily Gum Arabic (GA) supplementation on cardiovascular and metabolic risk factors and gastrointestinal symptoms in adults with or at risk of metabolic syndrome. | Risk factors for CVD, diabetes | Randomized controlled trial | Conducted between January and May 2018 | 80 Adults aged 18–50 with ≥2 risk factors for CVD | Volunteer recruitment from UAE University campus through advertising and interviews | Control group received 1 g/day of pectin (placebo) vs. 20 g/day of Gum Arabic for 12 weeks | 20 g/day GA powder (Acacia Senegal), administered in sachets, consumed with meals or beverages | Anthropometry, food diaries, IPAQ for physical activity, blood tests for metabolic markers, and GI/satiety questionnaires | The study measured changes in blood pressure, fasting blood glucose, HbA1c, lipid profile, body composition, energy and macronutrient intake, dietary fiber intake, bowel movement, and satiety levels. Outcomes were assessed at baseline and after 12 weeks of supplementation. | After 12 weeks, the Gum Arabic group showed significant reductions in systolic and diastolic blood pressure, fasting glucose, caloric and carbohydrate intake, and fat-free mass, along with a significant increase in dietary fiber intake and self-reported satiety. Improvements were also observed in bloating and bowel movements, though changes in BMI, lipid profile, and HbA1c were not statistically significant. The intervention group reported higher perceived appetite control and digestive comfort compared to controls. Overall, GA appeared to safely improve multiple cardiometabolic and gastrointestinal indicators. hese results suggest that GA could be a safe and beneficial adjunct to other treatments for those with, or at risk of, metabolic syndrome. | The sample size was relatively small and limited to a university-based population, affecting generalizability. The study lacked long-term follow-up, and the body composition assessment tool could not distinguish between fluid and lean tissue mass. Dietary and physical activity reporting were self-reported and may have introduced bias. Despite these, the study provides promising evidence supporting the role of GA in metabolic syndrome management. |
| Jarrar 2022 | Using Digital Platform Approach to Reduce Salt Intake in a Sample of UAE Population: An Intervention Study. | UAE | The aim of the study was to measure the effectiveness of using a digital platform approach (WhatsApp and electronic brochures) to deliver educational materials to facilitate salt reduction in a sample of the UAE population. ​ | Diet | Randomized controlled trial | The study started in October 2020 and lasted until January 2021, with a total duration of approximately 4 months. ​ The intervention itself lasted for 6 weeks, and measurements were taken at baseline and after 10 weeks. ​ | 121 Healthy individuals aged between 20 and 40 years | Participants were recruited using the following methods:  Email Circulation: Sent to non-medical students and staff from the Applied Sciences and Humanities Colleges at the United Arab Emirates University (UAEU). ​ Social Media Platforms: Recruitment was conducted through platforms like WhatsApp and Instagram to reach individuals outside UAEU. ​  Participants were randomly distributed into three groups (Control, WhatsApp, and Electronic Brochure) using the Altman and Bland procedure, with the RAND() function generated according to gender and age group. ​ | The study included three comparator groups:   Control Group: Consisted of 39 participants. ​ Did not receive any educational materials during the intervention period. ​ WhatsApp Group: Consisted of 41 participants. Received educational materials via WhatsApp messages every 7 days for 6 weeks, with each message repeated every 3 days. ​ Electronic Brochure Group:  Consisted of 41 participants. Received educational materials via email every 2 weeks for 6 weeks, with a new brochure sent each time. ​ | The study involved two intervention groups aimed at reducing salt intake using digital platforms:  WhatsApp Group: Participants received educational materials via WhatsApp messages. ​ A new message was delivered every 7 days for 6 weeks, with each message repeated every 3 days. ​Topics included the health hazards of high salt intake, the importance of checking food labels, and alternatives to salt. ​  Electronic Brochure Group: Participants received educational materials via email. ​ A new electronic brochure was sent every 2 weeks for 6 weeks, covering similar topics as the WhatsApp group.  The intervention lasted for 6 weeks, and its effectiveness was assessed using 24-hour urinary sodium excretion, potassium levels, creatinine levels, and a Knowledge, Attitude, and Practice (KAP) questionnaire. ​ | Data collection in the study was conducted using the following tools:  24-hour Urine Collection: Urine samples were analyzed for sodium, potassium, and creatinine levels  Knowledge, Attitude, and Practice (KAP) Questionnaire at baseline and after 10 weeks (including 6 weeks of educational intervention). ​ The questionnaire assessed knowledge related to salt and health outcomes, frequency of consumption, and perceived salt consumption. ​  Anthropometric Measurements: Body mass index (BMI)  Physical Activity was measured using the 7-day self-reported short International Physical Activity Questionnaire (IPAQ) at baseline and after the intervention. ​ | Key outcomes asseed were changes in salt intake as measured by reductions in 24-hour urinary sodium excretion; participants' knowledge, attitudes, and practices related to salt intake using a validated KAP questionnaire physical activity levels using the International Physical Activity Questionnaire (IPAQ), changes in practices such as adding salt during cooking, using table salt, and checking food labels for sodium content. ​ These outcomes were used to compare the effectiveness of WhatsApp messages and electronic brochures in reducing salt intake and improving KAP scores. ​  Another outcome was assessing the percentage of participants exceeding the World Health Organization (WHO) recommendations for sodium intake before and after the intervention. ​  The ultimate goal was to determine whether the UAE population is ready to adopt healthier practices regarding salt consumption and to provide insights for policymakers and the food industry to support salt reduction initiatives. ​ | Key Findings:  Salt Intake Reduction:  WhatsApp group: 278 mg reduction (p < 0.001)  Electronic Brochures group: 169 mg reduction (p = 0.018)  No significant change in the control group  Improved Compliance with WHO Guidelines:  WhatsApp group showed significant reduction (p = 0.023)  Overall compliance improved from 77.7% to 71.1% (p = 0.004)  Knowledge, Attitudes, and Practices (KAP):  Both intervention groups improved significantly in health knowledge and salt-reducing behaviors (p < 0.001)  Decreased salt use during cooking and increased food label reading  Behavioral Changes:  More use of salt alternatives (spices, herbs, lemon)  WhatsApp group showed greater shift toward buying low-salt foods (p = 0.002)  Intervention Effectiveness:  WhatsApp was more effective than brochures in improving outcomes  Potassium Intake:  Remained below WHO recommendations in all groups, highlighting a need to promote potassium-rich foods  Conclusion:  Digital tools, especially WhatsApp, are effective in reducing salt intake and improving dietary behaviors in the UAE. The population is receptive to health education with the right support. | Short duration (6 weeks) and no follow-up, limiting insight into long-term behavior change.  Narrow age range (20–40 years) and small, mostly Emirati sample, reducing generalizability.  Health outcomes (e.g., blood pressure) were not measured.  Relied on self-reported data, increasing potential bias.  Focused only on educational strategies, excluding broader interventions like food reformulation or policy changes.  Potassium intake was assessed but not promoted, missing an opportunity to address sodium–potassium balance. |
| Mussa 2019 | Personalized intervention to improve stress and sleep patterns for glycemic control and weight management in obese Emirati patients with type 2 diabetes: a randomized controlled clinical trial. | UAE | To evaluate the effectiveness of a personalized intervention targeting stress and sleep patterns on glycemic control and weight management among obese Emirati patients with type 2 diabetes. | Obesity, Type 2 Diabetes | Randomized controlled trial | Not explicitly stated; study involved a 25-week intervention period | 51 Emirati patients aged 18–60 with T2DM and BMI ≥25 kg/m², | Patients were recruited through clinic visits, flyers, and database contact; randomized into two groups | Intervention group received personalized stress/sleep management; control group received only assessments and general information | Personalized intervention included stress and sleep monitoring (via HRV), feedback, individualized action plans, and follow-up via in-person and phone sessions over 25 weeks | Heart rate variability (HRV) monitored with Firstbeat Bodyguard devices; anthropometric and biochemical parameters measured clinically; participants maintained diaries | Primary outcomes: Percentage changes in weight, BMI, and HbA1c. Secondary outcomes: Changes in stress and recovery levels, sleep duration and quality, and lipid profile indicators (HDL, LDL, TG, TC). | The intervention group showed significantly greater improvements than controls in weight (−3.2% vs −0.02%), BMI (−4.5% vs −0.0003%), and HbA1c (−5.3% vs +9.9%) over 25 weeks. There was a significant 84.5% increase in recovery levels and notable reductions in stress and improvement in sleep parameters in the intervention group, though not statistically significant for all metrics. Lipid profile changes (LDL, HDL, TG) favored the intervention group but did not reach significance. The study demonstrated that individualized stress and sleep-focused interventions could effectively improve both glycemic control and weight management in obese Emirati T2DM patients. he findings of the present study show that personalized approaches that reduce stress levels, increase recovery levels, and promote healthy sleep habits play an important role in weight management and glycemic control in T2DM | The small sample size (n=35 completers) limits generalizability and statistical power. The study’s short duration may have undercaptured longer-term outcomes. Stress and sleep effects varied among individuals, and although HRV offered objective monitoring, external lifestyle influences weren’t fully controlled. The absence of a placebo or blinding may also introduce bias. |
| Dalibalta 2017 | Exercise intervention on cardiovascular disease risk factors in a university population in the United Arab Emirates. | SHARJAH | Assess the effect of physical activity on cardiovascular disease (CVD) risk factors in a university population Investigate the effects of an 8-week exercise intervention on CVD risk factors | Physical activity | Non-randomized interventional study (Pre post study) | Start: April 2015 Duration: 8 months (April–December 2015) | 46 University students aged 19–23 years | Voluntary participation (details suggest convenience sampling among enrolled university students) | Control group: BMI < 25 (n=32) Experimental group: BMI ≥ 25 (n=14) | 8 weeks of moderate to high intensity exercise (60 mins, 3 times/week). Participants maintained exercise diaries for adherence. | Physiological: Seca scales, tape measures, VO2 max test, Pulsecor Cardioscope BP+ Biochemical: Reflotron Plus Clinical Chemistry Analyzer for cholesterol, triglycerides | Decrease in BMI, WHR Increase in HDL-cholesterol and VO2 max Mixed changes in blood pressure No significant changes in LDL or triglycerides | Significant improvement in cardiorespiratory fitness and HDL-cholesterol BMI and WHR decreased in both groups Systolic BP reduced slightly, especially in the control group No major change in total cholesterol, LDL, or triglycerides VO2 max improved from poor-fair to fair-good | Small sample size Short duration (8 weeks) Lack of dietary control Limited generalizability due to the university setting |
| Alkaabi 2021 | Effects of Diabetes Prevention Education Program for Overweight and Obese Subjects with a Family History of Type 2 Diabetes Mellitus: A Pilot Study from the United Arab Emirates. | Abu Dhabi | To assess the feasibility and effects of a diabetes prevention education program tailored to overweight/obese Emiratis with a positive family history of type 2 diabetes mellitus (T2DM), using the DiAlert protocol. | Multiple NCD diabetes, CVD, obesity | Non-randomized interventional study (Pre post study) | Data collection over a 6-month period | 32 Adults aged 18–55, Emirati national overweight or obese (BMI ≥25), and with at least one parent diagnosed with T2DM. | Convenience sampling through flyers and patient referral. Recruitment conducted near participants’ homes to reduce barriers. | Post-hoc grouping into:  “Responders” (≥2 cm reduction in waist circumference at 6 months)  “Non-responders” (<2 cm reduction) | Three individualized or family-involved interactive education sessions (baseline, 3, and 6 months), based on the DiAlert protocol. Included motivational interviewing, cultural tailoring, nutrition/exercise planning, and regular reminders (texts, videos). | Culturally adapted questionnaires on beliefs, risk perception, nutrition, self-efficacy  Anthropometry, blood pressure  Laboratory markers: HbA1c, lipid profile, creatinine, eGFR, vitamin D, inflammatory biomarkers (IL-6, TNF-α, hsCRP)  Accelerometer-based physical activity tracking (ActiGraph GT3X+) | Key outcomes measured was effectiveness of diabetes prevention program by measuring improvemnts in patients’ waist circumference, HbA1c, HDL, Serum creatinine, eGFR, Adiponectin, inflammatory markers and physical activity levels. Another key outcome measured was participant’s health bieliefs, risk perception about diabetes, nutrition and self-efficacy. | The diabetes education program was well accepted, with high retention and strong engagement. Although behavioral change (e.g., diet or physical activity habits) was limited, objective improvements in metabolic and inflammatory markers were noted, particularly in participants with reduced waist circumference. The program showed feasibility in terms of delivery and participant compliance. However, only ~25% of participants strongly internalized key health beliefs, underscoring challenges in health literacy and cultural engagement.  At 6 months: Significant improvements in: Waist circumference (−1.6%, p < 0.001), HbA1c (−2.3%, p = 0.007), HDL (+8.9%, p = 0.049), Serum creatinine (p = 0.025), eGFR (p = 0.009), Adiponectin (p = 0.024) 16/32 participants had ≥2 cm waist circumference reduction Physical activity improved more in responders (+66.1% vs. +7.4% in minutes/day of moderate-vigorous activity) Inflammatory markers (TNF-α, IL-6, hsCRP) decreased more in responders | Small sample size (n = 32)  No control arm  Short follow-up duration (6 months)  Uneven gender distribution (75% female)  High dropout from screening to enrollment  Findings not generalizable without larger RCTs |
| Hasan 2018 | Counting Footsteps with a Pedometer to Improve HMW Adiponectin and Metabolic Syndrome among Young Female Adults in the United Arab Emirates. | Sharjah | The aim of the study is to examine the effects of counting footsteps, using a pedometer, on high-molecular-weight adiponectin (HMW-Adip) and metabolic syndrome (MetS) components in normal and overweight/obese young female adults in the UAE. ​ | Physical activity | Non-randomized interventional study (Pre post study) | The study started on May 17, 2018, and lasted for approximately 9 weeks. | 60 Adult females aged 18–30 years.with normal or overweight/obese weight | Participants were recruited from the University of Sharjah, UAE. ​ The study used a quasi-experimental pretest-posttest design, and participants were divided into two subgroups based on their BMI (normal BMI and high BMI). Specific details about the sampling method (e.g., random sampling or convenience sampling) are not explicitly mentioned in the document. | The study included two comparator groups based on Body Mass Index (BMI):  Normal BMI Group: Participants with a BMI of 18–24.9 kg/m². ​ High BMI Group: Participants with a BMI ≥ 25 kg/m², indicating overweight or obese conditions. ​  These groups were compared in terms of their daily step counts, anthropometric measurements, and biochemical parameters before and after the 9-week intervention. ​ | The intervention involved participants wearing a pedometer (KenzLifeCoder e-step; Suzuken Company, Japan) throughout the day, except during bathing and sleeping, for nine consecutive weeks. ​ The pedometer counted their footsteps and estimated the calories burnt. ​ Participants recorded their daily steps and calories in a physical activity logbook at bedtime. ​Participants were asked to follow their routine activities and were encouraged to walk 10,000 steps per day. ​Weekly follow-up was conducted to ensure continuity and progress. ​ Devices were checked, and logbooks were collected. ​ Educational Material: Participants received educational material on the impact of increased body weight on health and lifestyle modification (dietary and physical activity). ​ A video link was shared to guide participants in achieving the goal of 10,000 steps per day. ​  The intervention aimed to estimate habitual physical activity and motivate participants to increase their daily step count. ​ | The study used the following tools and methods for data collection pre and post intervention:  Pedometer: Counted daily steps and estimated calories burnt – physical activity collected from logbook. ​  Anthropometric Assessments: Height, weight, BMI, body fat mass (BFM), percentage of body fat (PBF), visceral fat area (VFA), and fat-free mass (FFM). ​ Waist and Hip Circumference, Waist-Hip Ratio (WHR) using body composition analyzer, measuring tape and stadiometer.  Blood Pressure  Biochemical Parameters: Blood samples were collected after overnight fasting and tested for Total cholesterol (T-Chol), triglycerides (TG), high-density lipoprotein cholesterol (HDL-C), fasting glucose, insulin, LDL-C, Serum insulin and HMW-Adiponectin levels   Nutrient Intake: Assessed using a 24-hour diet recall method. ​ Nutrient analysis was performed using the ESHA nutrient analysis software   Metabolic Syndrome (MetS) Score: Calculated as the summation of the number of individual components of MetS present in each participant. ​ | Primary Outcome:  Change in high-molecular-weight adiponectin (HMW-Adip) levels. This was the main biomarker targeted to assess the physiological effect of increased physical activity using a pedometer.  Secondary Outcomes:  Physical Activity -Daily step count (measured via pedometer), anthropometric assessments, biochemical parameters, and metabolic syndrome score  Correlations and associations were also made bet  ween daily step count and outcomes like BMI, WHR, HMW-Adip, and MetS score | Over a 9-week period, participants averaged 7,056 ± 1,570 steps/day, with no significant difference between normal and high BMI groups. In the normal BMI group, significant reductions were seen in BMI, body fat mass (BFM), and waist-hip ratio (WHR). The high BMI group showed greater improvements, including significant reductions in BMI, BFM, percentage of body fat (PBF), waist circumference (WC), and visceral fat area (VFA).  Biochemical changes in the high BMI group included a decrease in triglycerides from 71.62 ± 29.22 to 62.50 ± 29.16 mg/dL (p = 0.003), reduced insulin levels from 21.7 ± 8.33 to 18.64 ± 8.25 µU/L (p = 0.046), and an increase in HMW-adiponectin from 3.77 ± 0.46 to 3.80 ± 0.44 µg/mL (p = 0.034). LDL-C levels also decreased significantly for all participants (p = 0.01).  Daily steps were inversely correlated with BMI (r = −0.33, p = 0.017), BFM (r = −0.29, p = 0.037), WHR (r = −0.401, p = 0.003), and MetS score (r = −0.49, p < 0.001), and positively correlated with HMW-adiponectin levels (r = 0.331, p = 0.017). No significant changes were found in dietary macronutrient intake.  Overall, the use of a pedometer significantly improved obesity markers, lipid profiles, insulin resistance, and adiponectin levels—especially in participants with higher BMI—highlighting its effectiveness as a motivational tool to reduce metabolic risk. | The study had several limitations that may affect the strength and generalizability of its findings. It used a quasi-experimental design without randomization and had a small sample size of only 52 participants, limiting causal inference and broader applicability. The short 9-week duration may not capture long-term effects, and the lack of a control group made it difficult to compare outcomes against a baseline or alternative intervention.  Participants self-reported their physical activity and calorie expenditure, introducing potential bias or inaccuracies. Although dietary intake was assessed, no dietary interventions were implemented, which could have influenced the results. The study population was limited to young females in the UAE, reducing generalizability to other groups.  Technical limitations included the use of a basic pedometer, which did not capture activity intensity or non-step activities, and the classification of physical activity based only on step count. Additionally, some biochemical markers (e.g., HDL-C) did not show significant changes, possibly due to insufficient duration or activity volume.  Overall, while the study offers useful insights, larger, randomized, and longer-term studies are needed to confirm its findings. |
| Ali 2021 | Feasibility Study of a Newly Developed Technology-Mediated Lifestyle Intervention for Overweight and Obese Young Adults. | UAE | The Rashakaty (Fitness for Me) study aimed to develop and test the feasibility of a technology-based nutrition education intervention that would support overweight and obese university students to achieve weight loss, enhance nutrition knowledge, and increase physical activity levels.. | Obesity | Non-randomized interventional study (Pre post study) | The study started in September 2016 and January 2017, with a duration of 16 weeks. | 246 Overweight or obese female students (BMI ≥ 25) aged 18-35 not involved in weight loss | Method of participant recruitment/sampling:  ​Participants were recruited using emails, university social media, and flyers in educational and residential campuses.  ​ Interested students registered on the program website and were scheduled for appointments via WhatsApp, phone calls, emails, and SMS text messages.  ​ | The study had two arms:  Rashakaty Basic (R-Basic): Implemented in the University of Sharjah, participants received access to a static website with educational material on healthier eating and physical activity.  ​ Rashakaty Enhanced (R-Enhanced): Implemented in the United Arab Emirates University, participants received a more comprehensive intervention based on social cognitive theory, including self-monitoring, goal setting, self-efficacy, problem-solving, and social support. | R-Basic: Access to a static website with educational material and questionnaires.  ​ R-Enhanced: Access to a dynamic intervention based on social cognitive theory, including self-monitoring tools (MyNetDiary app for diet tracking and PACER app for physical activity tracking), regular interactions with dietitians, goal setting, and social support.  ​ | Data was collected at baseline and 16 weeks after enrollment using:  Anthropometric Measurements: Height, weight, waist circumference, and body composition using bioelectric impedance.  ​ Nutrition Knowledge: General Nutrition Awareness Questionnaire (GNKQ).  ​ Physical Activity: International Physical Activity Questionnaire (IPAQ)—Short Form.  ​ Psychosocial Assessment: Health Beliefs Questionnaire adapted for university students in the UAE.  ​ | Key outcomes measured were the post-interventional comparisons between the R basic and R enhanced groups in changes in anthropometric data, nutrition knowledge, physical activity levels, perceived social support for healthier food choices and physical activity and self-efficacy to reduce sugar intake and overcome barriers to physical activity | The R-Enhanced group showed greater benefits than the R-Basic group across most outcomes. While both groups had no significant difference in weight loss, the R-Enhanced group had significant reductions in waist circumference, BMI, body fat percentage, and body fat mass.  In terms of nutrition knowledge, the R-Enhanced group improved across all assessed areas, while the R-Basic group improved only in dietary recommendations and food choices.  For physical activity, the R-Enhanced group saw broader gains, including increased vigorous and moderate activity, walking, and reduced sitting time. The R-Basic group only increased vigorous activity.  Regarding social support and self-efficacy, the R-Enhanced group reported improved support from both family and friends, and increased confidence in reducing sugar and overcoming physical activity barriers. The R-Basic group saw limited improvements, mainly in friend support for reducing fat and increasing fruit and vegetable intake. ​ | Short Duration: The intervention lasted only 16 weeks, which may not be sufficient to detect clinically significant weight loss.  ​ High Attrition Rate: A smaller sample of participants completed the endline assessments due to high attrition and unavailability during final exams.  ​ Baseline Differences: Significant differences at baseline between the two groups for some outcomes tested.  ​ Language Barrier: The website materials were not available in Arabic, which was preferred by participants.  ​ Participant Burden: Participants cited lack of time due to academic responsibilities, affecting their engagement in the program.  ​ Smartphone Requirement: Participation required a smart mobile phone, excluding those who did not use them.  ​ Cultural Relevance: Limited number of local foods in the diet tracking app and lack of apps in the Arabic language were barriers for participants. |
| Shehab 2016 | Evaluation and implementation of behavioral and educational tools that improves the patients' intentional and unintentional non-adherence to cardiovascular medications in family medicine clinics. | Abu Dhabi | To evaluate and implement behavioral and educational interventions that improve intentional and unintentional non-adherence to cardiovascular medications in patients attending family medicine clinics. | CVD | Non-randomized interventional study (Pre post study) | Conducted in 2010 Duration: 12 months | 300 Adult CVD patients >20 years old, UAE nationals | Random sampling from chronic disease registries of family medicine clinics | Not applicable (single-group pre-post intervention study) | Behavioral and educational tools based on the Theory of Planned Behavior and Health Belief Model targeting: - Patient factors (e.g., education, literacy)  - Provider communication  - Healthcare system issues (e.g., access, refill systems) Interventions reinforced every 3 months for 1 year | Validated Brief Medication Questionnaire (BMQ) (Arabic version)  Clinical parameters: blood pressure, FBG, PPBG, HbA1c, LDL  Data collected at baseline, 3, 6, 9, and 12 months | The primary outcome was the improvement in responses to BMQ scores (at 3, 6, 9 and 12 months after the interventions). The secondary outcomes were disease-related as fasting blood glucose (FBG), postprandial blood glucose (PPBG), glycated hemoglobin (HbA1c), low density lipoprotein-cholesterol (LDL-C) and blood pressure (BP). | Educational/behavioral interventions improved medication adherence in CVD patients  Adherence improved regardless of gender, with higher rates in university-educated and higher-income groups  Key barriers addressed: recall, belief/motivation, and access  Clinical outcomes significantly improved alongside adherence  No participant dropouts reported | Convenience sample from family medicine clinics; may not generalize to primary care or other settings  No control group  Self-reported adherence may introduce reporting bias  Results may not apply to non-UAE nationals or less structured clinics |
| Iskandar 2024 | Evaluating the influence of a 3-min online video on the community knowledge of stroke in four Arab countries. | MENA | To assess the impact of a short, evidence-based online educational video on public knowledge about stroke—including risk factors, warning signs, treatment, and prevention—across four Arab countries. | CVD | Non-randomized interventional study (Pre post study) | Conducted from July to September 2022 | 407 Adults (≥18 years) | Snowball sampling via university networks and social media (WhatsApp, Facebook, Instagram) | No control group; within-subject comparison of pre- and post-knowledge scores | A 3-minute online educational video on stroke (BE-FAST framework) was shown between a pretest and posttest | Structured online questionnaire (pretest/video/posttest) developed in English and Arabic, based on validated stroke education tools and guidelines (ASA, WHO) | The study measured changes in stroke knowledge scores before and after watching the video. Domains included stroke definition, types, risk factors, warning signs, prevention strategies, treatment options, and emergency response behavior (e.g., calling an ambulance). Country-specific knowledge levels, including in the UAE, were compared. Additionally, it examined factors affecting knowledge such as age, gender, education, profession, and health behaviors. | The video significantly improved overall stroke knowledge in all countries (mean score increased from 21.11 to 23.70, p<0.001). In the UAE, 407 participants showed a knowledge increase similar to the general trend (mean increase of +2.72 points). All knowledge domains—risk factors (e.g., obesity, diabetes, smoking), warning signs (e.g., paralysis, confusion), and emergency response—improved significantly (p<0.001). Those with lower baseline knowledge (non-health professionals, younger participants, unhealthy lifestyle) showed greater improvement. The video was effective due to its brevity, clarity, visual support, and bilingual accessibility. The findings highlight the utility of digital tools in stroke awareness, particularly in younger and educated populations. he educational tool successfully enhanced public understanding  of stroke risks, the identification of stroke signs, and the critical need for  emergency action. The advantages of this video include its short length, free  online access, use of evidence-based content in lay language, and reflective  images. The ultimate goal remains the long-term improvement of sustainability  by mandating full-scale trials | The study relied on a highly educated, internet-using sample, potentially limiting generalizability to the broader population, especially older adults or those with lower literacy. The absence of a control group and short follow-up period limit conclusions about long-term retention. Self-reported responses may introduce bias, and the online format prevented verification of individual engagement. The tool's sustainability and real-world health behavior impact require further longitudinal evaluation. |
| Jirjees 2024 | Time for health change: promoting community-based diabetes screening and prevention with video vignettes and social marketing. | UAE | To develop, validate, and evaluate a video-vignette to increase stakeholder understanding and engagement with a pharmacist-led diabetes screening and prevention service in the UAE, and to explore the video’s potential as a promotional and communication tool. | Type 2 Diabetes Mellitus | Non-randomized interventional study (Pre post study) | Exact start date not stated; video developed and validated over several months in 2023, with data collection extending into early 2024 | 1. Experts including HCPs 2. Overweight/obese adults aged 30 yrs and above  Script validation: 25  Video evaluation: 99  Focus groups/interviews: 22 Total = 146 participants | Purposive and convenience sampling; stakeholders recruited from pharmacies and clinics; interviews via Zoom | None — descriptive comparison between stakeholder categories (public, pharmacists, physicians) | Intervention is a proposed pharmacist-led diabetes screening and prevention service illustrated via a 3-segment scripted video showing:  Screening in a pharmacy  Referral to physician  Enrolment in a 6-month lifestyle change program | Script and video evaluation forms using 3-point and 7-point Likert scales  Focus group/interview guide based on the 4Ps of social marketing (product, price, place, promotion) | Study outcomes were to measure acceptance across stakeholder groups to assess for clarity, content, and value of video. These were measured by mean agreement scores (out of 7): Understanding the service, Realism and interest, Audio/visual quality. Through stakeholder engagement, the study assessed the public’s willingness to join screening programs, pharmacist’s enthusiasm to deliver such services, physicians opinions in expanding pharmacy roles. Participants also gave their suggestions for improvement and follow up. | The video-vignette was perceived as a compelling and engaging communication tool, significantly enhancing stakeholder understanding of the proposed diabetes screening and prevention service in pharmacies. Across physicians, pharmacists, and laypeople, the video scored highly for clarity, realism, and usefulness, and it sparked substantial interest in participation and support. Focus groups emphasized the power of visual storytelling to bridge knowledge gaps, and the format was clearly preferred over traditional written descriptions. The study contributes to growing evidence that multimedia, theory-driven communication tools can be instrumental in public health promotion, especially in culturally diverse settings like the UAE. | Real-world implementation of the service was not tested  The 3-point Likert scale for script validation lacked granularity  Cost of the service was not discussed in the video  Resource-intensive process for video development  Generalizability limited to similar settings where community pharmacy services are still evolving |
| Sadiya 2022 | Lifestyle intervention in early pregnancy can prevent gestational diabetes in high-risk pregnant women in the UAE: a randomized controlled trial. | Ajman | To evaluate whether a moderate-intensity, 12-week lifestyle intervention during early pregnancy could reduce the incidence of gestational diabetes mellitus (GDM) among high-risk women in the UAE. | Gestational Diabetes Mellitus (GDM) | Randomized controlled trial | Conducted between October 2018 and August 2020 | 63 Pregnant women aged 18–45, at risk of developing GDM | Recruited from the prenatal clinic at Sheikh Khalifa Hospital using computer-generated randomization in blocks of four | Usual Care group received standard antenatal care with general advice | 12-week moderate-intensity intervention involving dietary counseling, physical activity encouragement, and behavior modification, delivered via 2 face-to-face and 2 telephone sessions by licensed dietitians | 24-hour food recall, physical activity via mobile pedometer or self-report, OGTT at 24–28 weeks, antenatal clinic records | Primary outcome: Incidence of GDM at 24–28 weeks using IADPSG criteria. Secondary outcomes: Gestational weight gain (GWG), birth weight, cesarean section rate, and dietary/physical activity changes. | GDM occurred in 33.3% of the intervention group vs. 57.5% in usual care. Relative risk was 0.59 (p = 0.05); adjusted odds ratio for GDM was 0.26 (95% CI: 0.07–0.92, p = 0.04). The intervention group showed significant reductions in daily calorie (−120 kcal), carbohydrate (−19g), and fat intake (−5g), and increased physical activity (+52 min/week). Gestational weight gain was better controlled in the intervention group, with 93.4% gaining within IOM recommendations compared to 78.8% in UC. No significant differences were found in cesarean rate or neonatal outcomes, but adherence and feasibility of the intervention were high.  12-week moderate intensity lifestyle intervention in early pregnancy could reduce the relative risk of  GDM by 41% among high-risk pregnant women in the UAE. These findings could impact public health outcomes in  the region | The sample size was small and drawn from a single government hospital in Ajman, limiting generalizability. Dietary and physical activity data were self-reported, subject to recall bias. Pre-pregnancy weight was also self-reported. Blinding was not possible for participants or providers due to the nature of the intervention, though outcome assessors were blinded. Power was slightly below target due to limited recruitment. Nonetheless, the pragmatic design and high adherence enhance the study’s real-world relevance. |
| Farooqi 2022 | The Impact of Telemonitoring on Improving Glycemic and Metabolic Control in Previously Lost-to-Follow-Up Patients with Type 2 Diabetes Mellitus: A Single-Center Interventional Study in the United Arab Emirates. | Dubai | To evaluate the impact of telemonitoring (TM) devices, including home blood glucose and vital signs monitoring devices, on the glycemic control and compliance of previously lost-to-follow-up patients with type 2 diabetes mellitus (T2DM) | Type 2 Diabetes Mellitus | Non-randomized interventional study (Pre post study) | Not explicitly stated, but conducted for 3 months (follow-up period) per patient | 38 Type 2 DM patients Aged 18 yrs and above | Recruitment and Sampling: Retrospective review of Dubai Diabetes Center (DDC) databases to select patients with T2DM lost to follow-up. Study nurse contacted potential subjects via phone to check eligibility and willingness to participate. | No control group; single-arm interventional study design | The telemonitoring intervention provided patients with a comprehensive suite of home monitoring devices, including a OneTouch blood glucose monitor, electronic sphygmomanometer, heart rate monitor, pulse oximeter, and portable pill dispenser, along with a dedicated mobile phone equipped with the CHI app. After receiving standardized training, patients were instructed to perform daily data entry for three months, with readings automatically transmitted to clinic staff who could access the data via a dedicated laptop or the CHI app. Staff provided feedback and medical advice based on these readings, with the system generating automated reminders (averaging 2952 per patient). The intervention involved an initial clinic assessment where baseline data was collected and devices distributed, remote management during the three-month period, and a follow-up visit for outcome assessment, during which comprehensive measurements of HbA1c, ECG, pulmonary function, blood and urine analysis, and body composition were taken to evaluate the intervention's effectiveness. | Initial Visit: Demographics, medical history, medication history, current medications, body weight, vital signs, spirometry measurements, glycemic parameters, hemoglobin level, lipid profile, renal function tests, and urine analysis Telemonitoring Devices Provided: OneTouch Select Plus Flex blood glucose monitor, electronic sphygmomanometer, heart rate monitor and pulse oximeter, portable pill dispenser, and dedicated phone with CHI app preloaded Follow-up Visit: Conducted after three months, with same measurements as initial visit Center-based Measurements: HbA1c (BAYER DCA), ECG, pulmonary function, blood and urine analysis, and body weight/composition Home-based Measurements: Blood glucose, blood pressure, pulse rate, and oxygen saturation | Primary: Mean change from baseline in HbA1c level after three months of TM device use Secondary: Three-month changes in fasting and random blood glucose, body weight, blood pressure, pulse rate, oxygen saturation, spirometry measurements, hemoglobin, lipid profile, renal function, urine analysis, and ECG | Glycemic Control:  HbA1c decreased significantly from 10.3 ± 1.9% at baseline to 7.4 ± 1.5% after three months (mean difference: -2.9%, 95% CI: -3.6 to -2.2, p<0.001) 50% of patients (n=19) achieved HbA1c <7% after three months Fasting blood glucose decreased significantly by 40.1 mg/dL (95% CI: -70.8 to -9.3, p=0.013)  Metabolic Parameters:  Significant reduction in diastolic blood pressure (-3.5 mmHg, 95% CI: -6.6 to -0.4, p=0.028) Significant reduction in body weight (-1.3 kg, 95% CI: -2.5 to -0.08, p=0.037) Significant reduction in total cholesterol (-20.6 mg/dL, 95% CI: -33.9 to -7.3, p=0.003) Significant reduction in LDL cholesterol (-18.4 mg/dL, 95% CI: -29.5 to -7.3, p=0.002)  Device Utilization:  Mean usage days: Portable pill dispensers (86.5 ± 22.8 days), OneTouch blood glucose monitor (72.9 ± 23.5 days), electronic sphygmomanometer (62.3 ± 28.6 days), pulse oximeter (50.4 ± 28.6 days) Weak negative correlation between frequency of blood glucose monitor use and change in HbA1c (r=-0.028, p=0.866)  Safety: No adverse events reported by participants | Single-center setting and small sample size, which may limit generalizability No control group, preventing causal inference between TM implementation and diabetes control Short study duration (3 months) Did not investigate other factors that could interact with TM efficacy, such as educational level, socioeconomic status, and health literacy The study focused specifically on previously lost-to-follow-up patients, which may not represent the general T2DM population |
| Stanley 2017 | Implementation of a peer-mediated health education model in the United Arab Emirates: addressing risky behaviours among expatriate adolescents. | Abu Dhabi | To evaluate the efficacy of peer-mediated health education workshops in addressing risky behaviors (tobacco use, nutrition, and physical activity) among expatriate adolescents in Abu Dhabi, UAE. ​ | Multiple Risk factors: smoking, diet, Physical activity | Non-randomized interventional study (Pre post study) | Start Date: Not explicitly mentioned in the document. Duration: Pre- and post-workshop surveys were conducted approximately 2–3 weeks apart. ​ | 439 Expatriate Grade 9 students | Random sampling of private schools in Abu Dhabi. ​ Schools were contacted via phone calls, and the first 5 schools meeting the criteria and willing to participate were included. ​ Parental and minor consent forms were distributed and returned before scheduling the pre-workshop survey. ​ | Two groups were formed:  Tobacco Workshop Group: Focused on tobacco use. ​ Nutrition and Physical Activity Workshop Group: Focused on nutrition and physical activity. ​  Each group acted as a control for the other during analysis. ​ | Type: Peer-mediated health education workshops. ​ Duration: 45 minutes (1 class period). ​ Workshop Topics: Tobacco Workshop: Covered types of tobacco, health effects, influences on teen smoking, and benefits of cessation. ​ Nutrition and Physical Activity Workshop: Focused on healthy eating and exercise habits. ​  Implementation: Workshops were conducted by two trained university students (authors LWA and ZDS). ​ Materials included a workbook and interactive props (no PowerPoint presentations). ​ | Pre- and Post-Workshop Surveys: ​ Questions from the WHO Global Youth Tobacco Survey (GYTS) and CDC Youth Risk Behavior Surveillance System. ​ Surveys were anonymous and designed to assess knowledge, attitudes, and behaviors. | Outcomes were measured across the two separate intervention arms and their controls. The tobacco workshop group was assessed for  improvement in knowledge, attitudes, and perceptions about tobacco use compared to the control. The nutrition and physical activity workshop group was assessed for improvement in knowledge, attitudes, or perceptions about nutrition and physical activity and satisfaction with personal activity levels. ​ Peer-to-Peer Model Feedback was sought from participants. | Summary of Results: Tobacco Workshop: ​ Significantly improved knowledge, attitudes, and perceptions about tobacco use. ​ Positive changes were observed in 12 questions related to tobacco use, including correcting misconceptions about shisha and dokha smoking. Students reported healthier perceptions and attitudes toward tobacco cessation and the harmful effects of secondhand smoke.  Nutrition and Physical Activity Workshop: ​ No significant improvement in knowledge, attitudes, or perceptions about nutrition and physical activity. ​ Associated with decreased satisfaction with personal activity levels, indicating a need for alternative methods to address these topics. ​  Peer-to-Peer Model Feedback: ​ Majority of students found the workshops relatable, informative, and worth sharing with friends and family. ​ 83% of tobacco group and 70% of nutrition group planned to use the information for future health decisions. Students overwhelmingly recommended the peer-to-peer model for similar workshops. ​ | Self-Reporting Bias: ​ Data collection relied on self-reported surveys, which may be subject to social desirability bias, especially in the cultural setting of the UAE. ​  Survey Validity: The surveys used were not tested for reliability or validity in the UAE's cultural context. ​  Spillover Effects: ​ Tobacco workshop influenced responses to two nutrition and physical activity questions, suggesting potential overlap in workshop content.  Limited Scope: Study focused exclusively on 9th-grade students in private schools in Abu Dhabi with English curricula, excluding public schools, Arabic-language schools, and other grade levels. ​  Short Duration: ​ The study did not assess long-term behavioral changes due to the short time between pre- and post-workshop surveys. ​  Sample Size and Randomization: ​ Relatively small sample size and limited effectiveness of randomization in balancing the two workshop groups. ​  Exclusion of Emirati Perspective: ​ The study did not include local Emirati students, focusing only on expatriate populations. ​ Future studies should target local populations in their native language. |
| Ali 2024 | Impact of Skills for Change Program on metabolic control, diet and physical activity levels in adults with type 2 diabetes: A cluster randomized trial. | Abu Dhabi | To evaluate the impact of a culturally adapted 12-month lifestyle intervention program (Skills for Change) on glycemic control, diet, and physical activity levels among Emirati adults with type 2 diabetes. | Type 2 Diabetes Mellitus | Randomized controlled trial | Recruitment: November 2011 – January 2013 Intervention duration: 12 months | 382 Emirati national type 2 diabetes patients aged 20–70 years | Participants were recruited by physicians/nurses at AHS chronic disease clinics and via posters. Randomization occurred at the health center (cluster) level. | Intervention group received structured education and support; control group received standard diabetes care. | The “Skills for Change” program included seven individualized nutrition counseling sessions, five group sessions, group-based physical activity, behavior change strategies (self-monitoring, goal setting), educational videos, and printed resources. Control group received routine care only. | HbA1c and lipid profiles via blood samples  24-hour dietary recalls (Multiple Pass Method)  IPAQ-SF for physical activity  Anthropometric measurements (SECA scale and stadiometer)  SPSS for statistical analysis | The following key outcomes were measured pre and post intervention to compare the intervention group to control group: Change in HbA1c, BMI, Caloric intake, Nutrient intake,  Weekend sitting time, Physical activity improvements. | Although the intervention did not result in a statistically significant HbA1c reduction, it did lead to clinically meaningful improvements in body weight, caloric intake, and nutrient density of the diet, and reduced sedentary behavior on weekends. The program was especially effective in reducing BMI and promoting healthier eating. Implementation challenges included low attendance due to transportation and familial responsibilities, particularly among women. The authors highlight the importance of integrating culturally relevant strategies and expanding intervention intensity or delivery through digital platforms (e.g., WhatsApp). | Conducted only in Al Ain, limiting generalizability  No long-term follow-up post-intervention  Dietary and physical activity data were self-reported  Most participants were women and of lower educational attainment  Baseline HbA1c was <8%, limiting potential for significant reduction |
| El-Deyarbi 2024 | The effects of multifactorial pharmacist-led intervention protocol on medication optimisation and adherence among patients with type 2 diabetes: A randomised control trial. | UAE | To assess the impact of pharmacist-led multifactorial interventions on medication management optimization and patient adherence to medications | Type 2 Diabetes Mellitus | Randomized controlled trial | July 2021 to September 2022 (14 months) | 192 TYPE 2 diabetes patients between 30-65 years old | Patients were identified from endocrinology and chronic disease (CDC) outpatient clinics at Oud Al Touba Diagnostic and Screening Centre, Al Ain, UAE Simple randomization using computer-generated random numbers in Microsoft Excel with allocation concealment by sequential numbering Convenience sample determined by calculating a minimum sample size of 180 participants (82 per group with 10% dropout factored in) to provide 80% power with an alpha level of 5% | Intervention Group: Received the multifactorial pharmacist-led intervention protocol (MPIP) including medication adherence counseling and medication therapy management (MTM) Control Group: Received usual care (standard medication counseling) | Medication Adherence Counseling: 15-30-minute counseling sessions at initial and follow-up visits Barriers to adherence identified and addressed 15-minute follow-up phone interviews within two weeks of visits Adherence assessed using medication possession ratio and questionnaire  Medication Therapy Management (MTM): Comprehensive medication review and reconciliation Patient-specific medication booklet Training on SEHA mobile application for accessing medical records | Medication adherence measured using: Total medication possession ratio (MPRt) for all regimens Regimen-specific medication possession ratio (MPRs) Regimen non-persistence (RNP) Validated medication adherence questionnaire  MTM program outcomes Mobile application and medication booklet usage | Medication adherence (Total medication possession ratio (MPRt) for all regimens Regimen-specific medication possession ratio (MPRs) Regimen non-persistence (RNP))  MTM program outcomes Mobile application and medication booklet usage | Medication Adherence:  At 12 months, intervention group showed significantly higher overall medication adherence (MPRt of 0.95±0.09) compared to control (0.92±0.09), mean difference of 0.03 (95% CI 0.01-0.06, p=0.02) Similar improvements in regimen-specific adherence for antihyperglycemic, antihypertensive, and antihyperlipidemic medications Reduced medication non-persistence in intervention group (0.15±0.21) compared to control (0.22±0.23), p=0.02 Intervention group had higher adherence questionnaire scores: 70.2% had zero scores (high adherence) compared to 48.9% in control group  MTM Outcomes: 41 drug-related problems identified and 36 clinical interventions implemented in intervention group vs. 6 interventions in control group Medication optimization for 26 patients in intervention group vs. 4 in control group Medication substitution and discontinuation performed for patients in the intervention group  Mobile Application and Booklet Usage: Mobile application usage increased from 41.5% to 45.7% in intervention group vs. 21.4% to 19.3% in control group 27.6% of intervention group used medication booklet vs. 1% in control group | MPR may overestimate pill intake as some patients may refill prescriptions consistently but not adhere to medication Short study duration (12 months) with preset follow-up boundaries may underestimate compliance fluctuations SEHA mobile application was available to all participants and promoted by SEHA, potentially improving control group adherence Clinical outcomes were not set as primary outcomes Pharmacists were not blinded to groups during data collection and MPR estimation The cost-effectiveness of the intervention was not analyzed |
| Alzubaidi 2019 | Diabetes and cardiovascular disease risk screening model in community pharmacies in a developing primary healthcare system: a feasibility study. | UAE | To develop and assess the feasibility of a pharmacist-delivered screening model for diabetes and cardiovascular disease (CVD) risk in community pharmacies in the UAE. | Diabetes, CVD | Non-randomized interventional study (Pre post study) | Start: December 15, 2017 – May 8, 2018 (Approx. 5 months) | 115 Healthy adults Aged 40–74 years | Purposive sampling; pharmacist invitation in community pharmacies with promotional flyers | No comparator group – single-arm feasibility design | Pharmacist-led screenings: medical history, BP, BMI, HbA1c, cholesterol, ADA questionnaire Risk stratification: 10-year ASCVD risk ≥7.5%, HbA1c ≥5.7%, ADA score ≥5 → referred to physician | Paper-based record forms Point-of-care testing (Roche Cobas b 101 POC device) Omron BP monitor ADA risk questionnaire | Proportion of participants at high risk for diabetes/CVD Participant satisfaction with screening Feasibility of implementation in pharmacies | 57.4% referred for high risk 92.3% screenings completed in one visit 94.5% participants satisfied with service Screening duration: ~27 mins Only 24.3% visited physicians post-referral Lifestyle changes reported in >60% of those referred | Inability to verify follow-up with physicians due to fragmented healthcare system Short follow-up time Small sample, non-random selection Heavy reliance on pharmacist initiative for recruitment |
| Hazari 2023 | Effect of 8 weeks badminton session on cardiovascular and neuromuscular functions among older adults in United Arab Emirates: a quasi-experimental study. | Ajman | To analyze the effects of badminton on cardiovascular & neuromuscular function among older adults with and without non-communicable diseases in the United Arab Emirates | Physical activity | Non-randomized interventional study (Pre post study) | March 2022, 8 months | 120 patients aged 40 to 70 years, participants with non-communicable disease (diabetes mellitus, hypertension, and obesity only), age and gender- matched participants without the non-communicable disease and healthy control. | Purposive sampling method. The participants for the non-communicable group were approached through medical records and contacts at the Thumbay Hospital, Gulf Medical University. The participants in the health and without non- communicable disease were approached via flyers, and personal local contacts | Two interventional groups which consisted of participants with non-communicable disease (WCN, n=40), and participants without the non-communicable disease (WICN, n=40), and one non-interventional group (NIC) as healthy control participants were recruited. | The experimental group participants (WCN and WICN) were required to engage in the supervised Badminton game for 45-60 minutes per session, thrice a week for two months at mild to moderate exercise intensity, monitored on Rate of Perceived Exertion Scale (RPE 0-10). Participants in the NIC group were allowed to continue their daily routine activities as their regular choice and comfort. The age, gender, and level of participants were matched by the supervising physical therapist | The pre-participation screening questionnaire (PAR-Q) was administered to all participants to minimize the exercise-related risk and rule out any prior cardiovascular disorders before engaging in the badminton session. | The primary outcome variables and measures were focused on the cardiovascular, and neuromuscular components as listed below: Cardiovascular Parameters: ‐ 6-minute walk test: 6-minute walk distance (6MWD) and estimated VO2 peak ‐ Rate of Perceived Exertion (RPE) on 0-10 scale | The findings of the study indicated that there was a significant improvement in cardiovascular and many neuromuscular variables within and between the groups (p≤0.05) with maximum changes in participants with non-communicable diseases Engagement in sports like badminton can help to overcome the non- communicable disease burden. The immediate impact can be seen with the introduction of such interventional sports activities on a larger scale. Since the improvement was seen to be much better in the participants with non- communicable diseases, it could help to reduce the burden of non-communicable diseases. | Limitations include the relatively short duration of the intervention and the absence of a follow-up to assess long-term effects |

Supplementary Table 3: Characteristics of records based on grey literature (guideline/recommendations)

| **Title** | **Organisation** | **Publication date** | **Region where document applies to** | **Brief summary** | **NCD addressed** | **Document type** | **Target population** | **Key Outcomes** |
| --- | --- | --- | --- | --- | --- | --- | --- | --- |
| Scope of Practice and Clinical Responsibilities of Family Medicine | DHA | 2016 | Dubai | Guidelines on scope of practice and responsibilities of family physicians towards mangment of diabetes, CVD and obesity | Cardiovascular diseases, chronic illnesses, nutrition-related conditions | Guideline | HCPs | Family physicians, as outlined by DHA, are responsible for:  Diabetes: Early identification, diagnosis, complication screening (e.g., retinopathy), chronic disease monitoring, and managing emergencies like DKA.  Obesity: Assessing contributing factors, interpreting BMI, advising on diet and physical activity, and recognizing obesity as both a disease and risk factor.  Cardiovascular Disease: Identifying risk factors, using clinical tools (e.g., BP, ECG, lab tests), managing heart failure exacerbations, educating patients on risk reduction, and leading primar and secondary prevention efforts through a team-based, holistic approach. |
| HAAD Guidelines for the Provision of Cardiovascular Disease Management Programs | HAAD | 2017 | Abu Dhabi | Guidelines for Cardiovascular Disease management programs that treat those identified from the Weqaya screening program to be at high risk of cardiovascular disease. The guideline highlights recomendations for registered healthcare facilities and healthcare providers on patient assessment, risk scoring, patient care, performance reporting, and data collection. | Cardiovascular diseases (CVD) and related risk factors | Guidelines | HCPs, facilities, secondary are the patients | Disease Management Programs (DMPs) including screening, early diagnosis, lifestyle interventions (e.g., diet, physical activity, tobacco control), and evidence-based healthcare interventions |
| UAE National NCD Action plan 2017-2021 | MOHAP | 2017 | UAE | UAE's strategic approach to addressing noncommunicable diseases and their risk factors- General framework for determinants and controls that are used in formulating policies, developing legislation, and estimating material resources to support inputs for prevention programs from these diseases | Cardiovascular diseases, diabetes, obesity | National strategic goals | General Public | The plan outlines a multisectoral approach for preventing non-communicable diseases, Key strategies include strengthening legislation to reduce risk factors (tobacco, unhealthy diet, physical inactivity), implementing early intervention programs starting in childhood, and ensuring equitable access to primary healthcare. The plan also prioritizes community-based interventions, early detection and screening programs, and building a robust surveillance system to monitor progress.It emphasizes community participation, respecting cultural differences, and ensuring adequate financial and technical resources for effective implementation with measurable results.  Objectives: to reduce premature deaths from major non-communicable diseases by 25% through comprehensive targets including stopping obesity/diabetes increases, reducing physical inactivity, hypertension, salt/fat consumption, and smoking rates, while ensuring 90% availability of diagnostic services and essential medications at primary healthcare centers by 2025 |
| UAE National Health Survey Report 2017-2018 | MOHAP | Not specified | UAE | Report on burden of three respective NCDs and risk factors among other health conditions. | Cardiovascular disease, diabetes, risk factors | Report |  | It highlighted the self-reported prevalence rates of the three NCDs, among both nationals and non-nationals. It also assessed the health behaviours and prevalence of risk factors including use of tobacco, healthy eating and physical activity. Clinical and Biochemical Measurements including BMI, waist circumference , blood pressure levels.as well as health expenditures were also reported |
| Triage Protocol for Hyperacute Stroke Emergencies and their Referrals in Pre-Hospital and Emergency Department (ED) Setting: EMS and Self-Presenting Emergency Departments’ Arrivals | DOH | 2017 | Abu Dhabi | Guidelines on the process of the triage process that Emergency Medical Services (EMS) and all healthcare facilities must follow for both EMS-driven and self-presenting hyperacute stroke cases | Cardiovascular disease | Guidelines | Health care professionals | The protocol outlines clear process for all healthcare facilities and Emergency Medical Services (EMS) in Abu Dhabi to follow for both EMS-driven and self-presenting hyperacute stroke cases. This protocol aims to ensure that no such emergency case is rejected, regardless of insurance coverage , and that patients are transferred and treated at the most appropriate facility as quickly as possible. The protocol also specifies criteria for hospitals to be designated as a "Comprehensive Stroke Centre" |
| DOH service requirements for the weight management program for overweight and obese children | DOH | 2018 | Abu Dhabi | Comprehensive clinical guideline for healthcare facilities implementing weight management programs for children in Abu Dhabi. | Obesity and related metabolic disorders | Guidelines | HCP, Overweight and obese children | The DOH outlines a structured, multidisciplinary weight management program for overweight and obese children aged 2–18 in Abu Dhabi. It includes lifestyle, pharmacological, and surgical interventions when needed. The program operates at two levels: primary care for standard cases and specialized centers for complex cases. It uses WHO/CDC growth charts for diagnosis, includes screening for comorbidities, and sets age- and condition-specific weight goals. The program emphasizes evidence-based care, defines outcome measures, and provides guidance on insurance coverage for both nationals and non-nationals. |
| Dubai Household Health Survey | DHA | 2019 | Dubai | Report on the findings of the 2019 Dubai Household Health Survey (DHHS) reflecting the health situation in the Emirate | Diabetes, CVD, Risk factors | Report | General Public | It highlighted the self-reported prevalence rates of hypertension, diabetes, angina stroke, those on medication, those in need of services, including both nationals and non-nationals. It also assessed the health behaviours and prevalence of risk factors including use of tobacco, healthy eating and physical activity. Clinical and Biochemical Measurements including BMI, waist circumference , blood pressure levels. All population groups were addressed including migrant labor groups. Health expenditures were also reported |
| The Case for Investment in Prevention and Control of Non-Communicable Diseases in the United Arab Emirates | MOHAP, UNDP, WHO | 2021 | UAE | Economic investment case analysis for addressing non-communicable diseases (NCDs) in the UAE. It examines direct and indirect costs associated with NCD burden and costs for implementation of interventions over a 15-year period (2020-2034). | Cardiovascular disease, diabetes, risk factors | Report | Policy makers. | Cardiovascular disease represents the largest component of the UAE's NCD economic burden. The analysis demonstrates that investing in these four evidence-based "best buy" intervention packages can deliver significant reductions across all major NCDs - cardiovascular disease, cancer, chronic respiratory disease, and diabetes - while providing strong economic returns that justify the upfront investment costs. Key recommendation sinclude to invest an scale up in salt control and tobacco reduction, increase taxation on harmful products, engage multiple sectors and to test innovative approaches |
| EJADA Program DIABETES KPIs and Recommendations | DHA | 2023 | Dubai | Guidelines on managment of diabetes and risk factors | Diabetes Type two and pre diabetes | Recommendations | HCPs | Pharmacological: Includes medications like metformin, GLP-1 receptor agonists, SGLT2 inhibitors, and more. Non-Pharmacological: Lifestyle interventions (e.g., diet, exercise), referrals to specialists like endocrinologists, psychologists, and dietitians, Screening, diagnosis, periodic monitoring, and prevention of diabetes progression. |
| Standards for Bariatric Surgery Services | DHA | 2023 | Dubai | A DHA circular highlighting the required standards for DHA-approved bariatric surgery services including registration and license procedures, health facility requirements, healthcare professional requirements, pre-operative evaluation and informed consent, bariatric surgery services, critical support and continuity of care. | Obesity and related metabolic disorders | Guidelines | HCPs | Includes bariatric surgery, pre-operative and post-operative care, multidisciplinary team involvement, and patient education |
| Obesity Study, 2023 | MOHAP | 2023 | UAE | Prevalence of obesity (BMI ≥ 30), gender-specific and nationality-specific obesity rates, and bariatric surgery outcomes | Obesity and related health conditions | Report from prevalence research | Not specfiied | Highlights include obesity prevalence rates (e.g., 27.8% overall in UAE, with variations by emirate) and bariatric surgery statistics (e.g., 13,484 surgeries over three years) |
| School Screening Standard | DOH | 2023 | Abu Dhabi | Describes guidleines of annual and comprehensive school screenings, including BMI, vision, hearing, physical exams, and health education | Health conditions identified through school screenings | Recommendation | School admin, school nurse (HCP?). School children | Describes guidleines of annual and comprehensive school screenings, including BMI, vision, hearing, physical exams, and health education |
| Acute Stroke Centers Inspection Checklist – Random | DHA | 2024 | Dubai | Checklist for requirement for acute stroke centers to ensure high quality service | Stroke and related cerebrovascular diseases | Guideline | HCPs, Health care facilities | Prevention, early detection, treatment, rehabilitation, emergency care, and continuous follow-up |
| EJADA Program Obesity and metabolic syndrome KPIs and Recommendations | DHA | 2024 | Dubai | Guidelines on diagnosis and managent of obesity | Obesity and metabolic syndrome | Recommendation | HCPs | Includes radiodiagnosis, pharmacological interventions (e.g., GLP-1 receptor agonists), bariatric surgery, and referrals to specialists like endocrinologists, nutritionists, and psychologists |
| Dubai Periodic Health Screening Guidelines | DHA | 2024 | Dubai | Recomendation for an evidence based screening program for adults in Dubai that covers screening for cardiovascular disease, diabetes, hypertension, cancers, osteopororsis and mental health disorders. | Cardiovascular diseases, diabetes, kidney disease | Recomendations | HCPs | Early detection, periodic screening, lifestyle interventions, pharmacological treatments, and referrals to specialists |
| Standard for Diagnosis and Management of Diabetes Mellitus Type 1 and 2 | DOH | 2024 | Abu Dhabi | Guidelines on Screening, diagnosis, treatment, lifestyle interventions, pharmacological therapies, continuous glucose monitoring (CGM), and self-management education | Diabetes Mellitus (Type 1, Type 2, gestational diabetes, and prediabetes) | Recommendation | HCPs | Guidelines on Screening, diagnosis, treatment, lifestyle interventions, pharmacological therapies, continuous glucose monitoring (CGM), and self-management education |
| Tobacco and Electronic Smoking Control Program | ADPHC | 2024 | Abu Dhabi | Advocacy for a smoke-free environment, implementation of Federal Law 15 of 2009 and Executive By-law 24 of 2013, awareness campaigns with the slogan "Together Towards a Smoking-Free Abu Dhabi", and smoking cessation services at clinics across the region | Cardiovascular diseases, chronic respiratory diseases, cancers, and hypertension | Program | General public | Detailed information about various tobacco products (including cigarettes, shisha, medwakh, and e-cigarettes), their health impacts, strategies for quitting, and a directory of smoking cessation clinics available throughout Abu Dhabi, Al Ain, and Al Dhafra regions to support those seeking to quit tobacco use. |
| Diabetes prevention | ADPHC | 2024 | Abu Dhabi | Provides general information about diabetes Type 2 and including risk factors and diagnostic criteria. Advises on prevention strategies and available screening services in Abu Dhabi. | Diabetes Type 2 | Program | General public | Explains what diabetes is and how it develops, detailing the four main types (Type-1, Type-2, Gestational, and Impaired Glucose Tolerance), with a focus on Type-2 diabetes which affects approximately 95% of diabetic patients worldwide. The guide outlines both modifiable risk factors (weight, diet, physical activity, tobacco/alcohol use) and non-modifiable risk factors (ethnicity, family history, age, gender), common symptoms, diagnostic criteria according to WHO guidelines, and potential complications of uncontrolled diabetes. It emphasizes prevention strategies including weight management, healthy eating, regular physical activity, and regular screening through the Comprehensive Screening Program (IFHAS), which is available to Thiqa insurance cardholders at various healthcare facilities throughout Abu Dhabi. |
| Standard for Non-surgical Management of Obesity | DOH | 2025 | Abu Dhabi | Guidelines for implementing comprehensive obesity management programs within healthcare facilities | Obesity and related metabolic disorders | Guidelines | HCPs, Children 2-18 years, adults | The document outlines: a risk-stratified approach based on BMI, waist circumference, and the Edmonton Obesity Staging System Comprehensive screening and assessment protocols Multi-component interventions combining diet, exercise, behavioral modification, and when appropriate, pharmacotherapy Specific criteria for pharmacological interventions, including GLP-1 receptor agonists, lipase inhibitors, and combination medications Requirements for multidisciplinary teams (physician, dietitian, exercise physiologist, behavioral therapist, nurse) |
| DHA telehealth clinical guidelines for virtual management of obesity | DHA | 2024 | Dubai | Clinical guidelines for the virtual management of obesity through telehealth services | Obesity | Guidelines | Healthcare professionals. patients | It covers the guidelines for, clinical assessment, diagnostic considerations, evaluation methods, and treatment approaches for obesity management via telephone or video consultations. The guidelines emphasize evidence-based practice and include detailed protocols for risk stratification, lifestyle interventions, pharmacological therapy, medical devices, and bariatric surgery considerations. |
| DHA telehealth clinical guidelines for virtual management of type 2 diabetes mellitus | DHA | 2021 | Dubai | Clinical guidelines for the virtual management of diabetes through telehealth services | Diabetes | Guidelines | Healthcare professionals. patients | This guideline provides evidence-based protocols for virtual management of Type 2 diabetes, enabling healthcare providers to safely diagnose, treat, and monitor diabetic patients through telehealth platforms. It outlines early metformin use, systematic medication escalation, aggressive management of cardiovascular risk factors, and regular monitoring of key parameters |
| DHA telehealth clinical guidelines for virtual management of hypertension | DHA | 2024 | Dubai | Clinical guidelines for the virtual management of hypertension through telehealth services | Hypertension | Guidelines | Healthcare professionals. patients | It covers the guidelines for the virtual management of hypertension through telehealth services, covering evidence-based diagnosis, staging (Stage 1 and Stage 2 hypertension), laboratory monitoring requirements, non-pharmacological and pharmacological treatment approaches, referral criteria, and red flags for emergency situations. The document emphasize proper blood pressure measurement techniques, risk assessment, lifestyle interventions, and appropriate medication management via virtual consultations. |
| UAE National Health Survey Summary Report for Elderly Respondents (60+) | MOHAP | 2025 | UAE | Report on burden of three NCDs in the elderly aged (60+ years) people | Diabetes, CVD, Cholesterol | Report | Adults aged above 60 years | The report assesses the burden of the three NCDs by investigating the rate of participants, diagnosed with diabetes, stroke, hypertension, hypercholesterolemia, those taking medication and those on herbal remedies for the diseases. It provides information on Clinical and Biochemical Measurements included BP, BMI, impaired glucose levels, cholesterol levels |
